# Previous Motor Actions Outweigh Sensory Information in Sensorimotor Statistical Learning

**DOI:** 10.1523/ENEURO.0032-21.2021

**Published:** 2021-09-28

**Authors:** Barbara Feulner, Danilo Postin, Caspar M. Schwiedrzik, Arezoo Pooresmaeili

**Affiliations:** 1 Perception and Cognition Lab, European Neuroscience Institute Göttingen-A Joint Initiative of the University Medical Center Göttingen and the Max-Planck-Society, Göttingen 37077, Germany; 2 Bioengineering Department, Imperial College London, London SW7 2BU, United Kingdom; 3 Department of Psychiatry, School of Medicine and Health Sciences, Carl von Ossietzky University of Oldenburg, Bad Zwischenahn 26160, Germany; 4 Neural Circuits and Cognition Lab, European Neuroscience Institute Göttingen-A Joint Initiative of the University Medical Center Göttingen and the Max-Planck-Society, Göttingen 37077, Germany; 5 Perception and Plasticity Group, German Primate Center–Leibniz Institute for Primate Research, Göttingen 37077, Germany; 6 Leibniz ScienceCampus Primate Cognition, Göttingen 37077, Germany

**Keywords:** learning, prior, probabilistic, representation, saccade, sensorimotor

## Abstract

Humans can use their previous experience in form of statistical priors to improve decisions. It is, however, unclear how such priors are learned and represented. Importantly, it has remained elusive whether prior learning is independent of the sensorimotor system involved in the learning process or not, as both modality-specific and modality-general learning have been reported in the past. Here, we used a saccadic eye movement task to probe the learning and representation of a spatial prior across a few trials. In this task, learning occurs in an unsupervised manner and through encountering trial-by-trial visual hints drawn from a distribution centered on the target location. Using a model-comparison approach, we found that participants’ prior knowledge is largely represented in the form of their previous motor actions, with minimal influence from the previously seen visual hints. By using two different motor contexts for response (looking either at the estimated target location, or exactly opposite to it), we could further compare whether prior experience obtained in one motor context can be transferred to the other. Although learning curves were highly similar, and participants seemed to use the same strategy for both response types, they could not fully transfer their knowledge between contexts, as performance and confidence ratings dropped after a switch of the required response. Together, our results suggest that humans preferably use the internal representations of their previous motor actions, rather than past incoming sensory information, to form statistical sensorimotor priors on the timescale of a few trials.

## Significance Statement

Humans can learn statistical regularities and later use them as priors to inform decisions. It remains unclear what type of representation is used to store and integrate past experience. We designed an experiment where humans had to combine visual information over multiple trials to locate a hidden target location. Using computational modeling, we found that participants represented past experience in the form of their previous decisions, and not directly by memorizing the visual cues. As a consequence of overweighing past decisions relative to the veridical visual information, gained experience did not generalize across two different contexts, albeit they differed minimally with respect to the prior. Hence, the process through which past experience is learned determines its influence on our decisions.

## Introduction

We often have to make decisions based on sparse and uncertain sensory information. Previous research has shown that in these cases humans use Bayesian inference where the current sensory information (likelihood) and the previously acquired knowledge (priors) are integrated, each weighted by their respective uncertainty (Körding and Wolpert, 2004; Wei and Körding, 2010). While the majority of previous studies have examined whether the perceptual and sensorimotor decisions follow the rules of a Bayesian framework (Ernst and Banks, 2002; Körding and Wolpert, 2004), less emphasis has been placed on understanding how likelihoods and especially statistical priors are learned and represented in the first place. A number of elegant recent studies have tried to bridge this gap by investigating how people learn likelihoods (Sato and Kording, 2014) and priors (Berniker et al., 2010) to perform Bayesian computations. Interestingly, the timescale of the two types of learning varied vastly, with fast learning of likelihood but slow learning of prior distributions. It remains unknown why such an asymmetry should exist, as theoretically both types of learning are equivalent. It has been hypothesized that learning about the likelihood versus learning about the prior involves different neural mechanisms, potentially hinting to the fact that their respective distributions might be represented in different regions of the brain (Vilares et al., 2012) .

Learning of statistical priors is itself not a uniform process as it shows dependencies on the specific context where the learning occurs. In the Bayesian framework, priors are a form of abstract knowledge (Tenenbaum et al., 2006, 2011; Battaglia et al., 2013), which can be generalized across different contexts. However, previous findings regarding the generalization of statistical priors have been mixed. Some studies have shown that statistical learning of priors is very narrow-band and context/modality specific in perceptual (Frost et al., 2015) and sensorimotor (Hewitson et al., 2018; Yin et al., 2019) domains, thus preventing learned information to transfer to different contexts/modalities. Other studies, on the other hand, provided evidence for generalization (Sato and Kording, 2014; Kiryakova et al., 2020), although generalization, in some instances, seemed to occur differently for different parameters of a statistical distribution, e.g., the mean and the variance of a distribution (Fernandes et al., 2014). The finding that some aspects of learning could generalize, while others could not, was confirmed by a recent study showing that, for instance, in Bayesian time estimation, priors can be generalized across stimuli but not motor actions (Roach et al., 2017).

Therefore, despite an increasing number of studies testing generalization and transfer, the exact rules determining generalizability remain unclear. One potential reason for these seemingly contradictory results is a lack of a formal definition of what is learned. It has been argued that when learning is not generalized, a policy (i.e., a specific rule for action) rather than knowledge (i.e., abstract and context-independent information) is acquired through learning (Chambers et al., 2019). However, it is not clear what features of the learning dynamics determine whether a policy or knowledge is acquired during encounters with the learned information.

To investigate learning and generalizability of a prior distribution, we employed a saccadic eye movement task similar to the design of a previous study (Dekleva et al., 2016), where participants had to learn to locate a hidden target. The location of the hidden target corresponded to the mean of a circular normal distribution. In each trial, a visual hint sampled from the underlying distribution was shown and participants indicated their current estimate with a saccadic eye movement, looking either toward (pro-saccade) or to the exact opposite direction of the estimate (anti-saccade). To successfully estimate the hidden target location, participants had to combine information across multiple trials. This design allowed us to investigate whether participants formed their prior knowledge by combining the visual information or by combining the previous motor actions across time, under different saccadic response contexts. Our results from two experiments indicate that sensorimotor learning of a spatial prior in both response contexts is largely guided by previous motor plans, rather than by previous sensory input in form of visual hints. Despite the high degree of similarity of pro-saccades and anti-saccades in their learning behavior, suggesting a motor-independent learning algorithm, the learned prior in one context did not generalize to the other. We propose that the lack of transfer between the two contexts is a natural consequence of their shared learning algorithm in which previous motor actions outweigh sensory information.

## Materials and Methods

In this study, we report the results of two experiments investigating how a spatial prior is learned under different oculomotor response contexts. The second experiment was identical to the first and served as a control for ensuring that participants were aware of how the location of the learned prior varied across blocks of the experiment (see the description of experiment 1 and experiment 2). We therefore describe the methods common to both experiments and mention the differences where they apply.

### Participants

In total, 41 participants were recruited for this study; 21 participants (age range 22–38 years, main = 26.05, SD = 3.80, 10 females) took part in the first experiment. All participants had normal (*N* = 9) or corrected-to-normal vision (*N* = 12). One participant was excluded from the data of the first experiment as the postexperiment questionnaire indicated that the participant had misunderstood the task. A total of 20 participants (age range 23–38 years, main = 28.95, SD = 5.07, 11 females) took part in the second experiment (with no exclusion) and all had normal (*N* = 16) or corrected-to-normal vision (*N* = 4). Participants were recruited from the general population of the city of Göttingen, Germany, using flyer and online advertisement and received cash financial compensation for their participation. Participation was voluntary; all participants were informed about the study procedure and gave written consent before the test session. The study was approved by the local ethics committee of the Universitätsmedizin Göttingen (UMG), under the proposal number 15/7/15.

### Experimental setup

The stimuli were presented at the center of a calibrated ViewPixx/EEG monitor (VPixx Technologies; dimension: 53 × 30 cm, refresh rate: 120 Hz) with a resolution of 1920 × 1080 pixels at a viewing distance of 60 cm. All experiments were scripted in MATLAB, using Psychophysics toolbox (Brainard, 1997). Eye movements were measured using the Eyelink1000+ eye tracking system (SR Research) in a desktop mount configuration, recording the right eye, with a sampling rate of 1000 Hz. A chin rest was used to stabilize the participant’s head. The EyeLink camera was controlled by the EyeLink toolbox in MATLAB (Cornelissen et al., 2002). At the beginning of each experiment, as well as after every 10 blocks of the hidden target task (see experiment 1), the eye tracking system was calibrated using a 13-point standard EyeLink calibration procedure. Calibration was repeated until an average error of maximum 0.5 degrees of visual angle (dva) was achieved, and the error of all points was below 1 dva. If the calibration accuracy dropped during the experiment, e.g., because of the subjects’ movement, the experimenter recalibrated the eye tracking system again.

### Experiment 1

**Figure 1.**
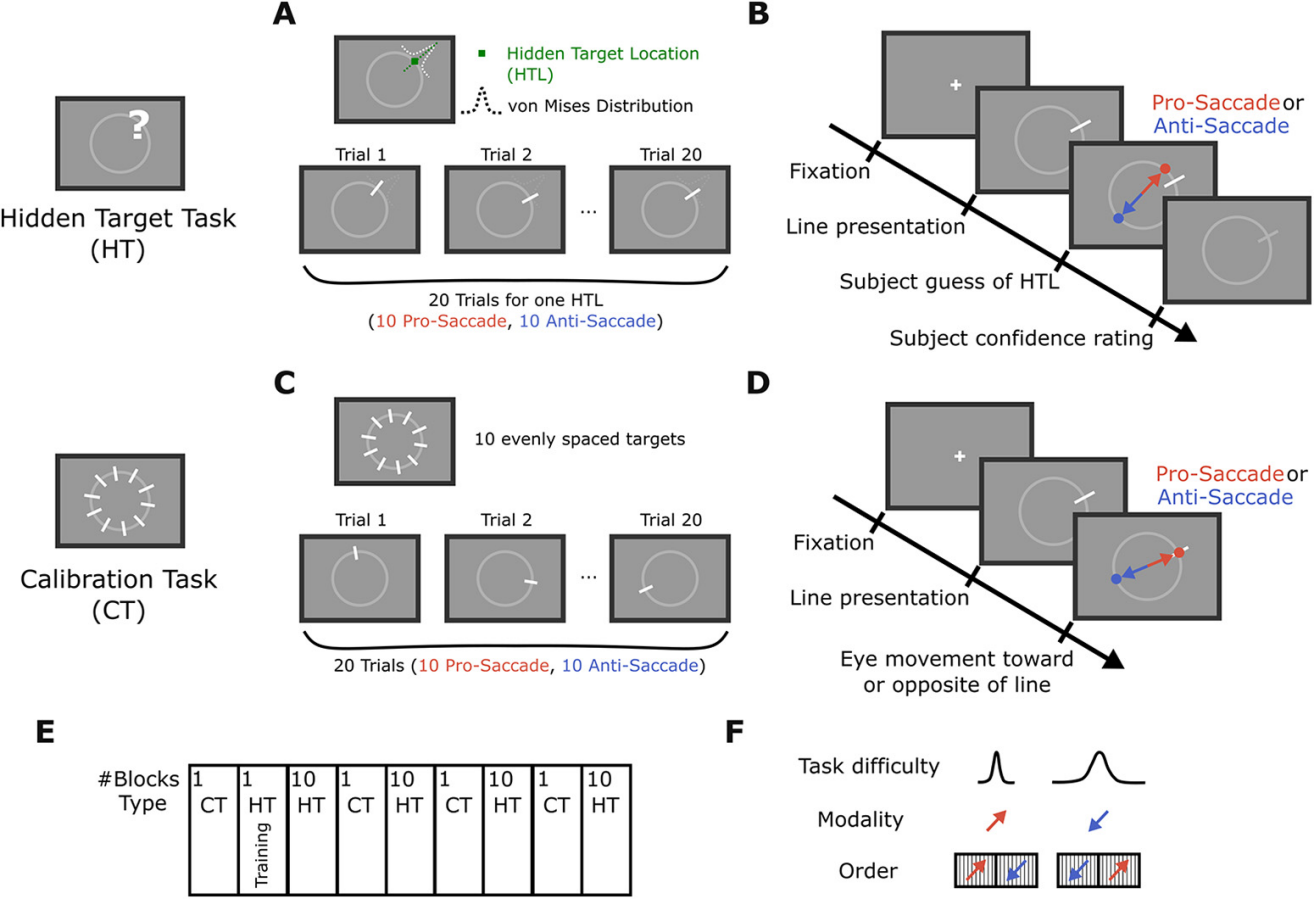
Experimental design of the hidden target task employed to study the statistical learning of a spatial prior in two different visuo-motor contexts. ***A***, ***B***, Main task of the experiment. ***A***, Participants were told to estimate the location of a hidden treasure on a ring by observing and combining information provided by the visual hints across trials. The hidden target location was defined as the mean of a von Mises distribution and the hints, presented at each trial, were samples drawn from this underlying distribution. Participants had 20 trials to estimate the location of the hidden target, after which a new hidden target had to be found. Participants used their gaze to indicate their responses. ***B***, Each trial started with a fixation period, after which the hint was presented, and participants had to indicate their guess about the location of the hidden target by either looking at it (pro-saccade response) or by looking exactly opposite to it (anti-saccade response). In half of the trials (i.e., consecutive 10 trials), participants had to use pro-saccades, and in the other half they used anti-saccades, with a randomized order across blocks. ***C***, ***D***, Calibration task used to estimate the motoric error of each participant for pro-saccades and anti-saccades. Participants had to directly look either at the lines (pro-saccade response) or exactly opposite to the lines (anti-saccade response). ***E***, Block-design of the experiment. ***F***, We compared learning across two levels of difficulty and two different response types. Task difficulty was varied by changing the concentration of the von Mises distribution (compare Materials and Methods). Finally, we tested whether knowledge could be transferred from one visuo-motor context to the other. For this, we also varied the order of pro-saccade and anti-saccade responses across blocks.

The experiment comprised two tasks: a calibration task (to estimate the motor error of pro-saccades and anti-saccades) and the main task, referred to as the “hidden target task” (Fig. 1). There were in total four blocks of the calibration task (*n* = 20 trials in each block) and 40 blocks of the hidden target task (*n* = 20 trials in each block). Each experiment started with a block of the calibration task followed by one training block for the hidden target task (*n* = 10 trials in this block). The data from this training phase was not analyzed. Thereafter, the experiment proceeded to the main task where participants performed 10 blocks of the hidden target task followed by one block of the calibration task. This sequence was repeated four times (Fig. 1*E*).

#### Hidden target task

Participants were instructed to look for a “hidden treasure” location on a ring with a radius of 7.5 dva, centered in the middle of the screen (Fig. 1*A*). The word hidden treasure was used in our instructions to the participants to make the task more realistic and engaging, however we will refer to the task as the hidden target task throughout. Each trial started with a fixation period, where participants had to fixate for 0.5 s on the white cross (size = 0.1875 dva, color: white, displayed on a half-gray background) in the middle of the screen (Fig. 1*B*). After that, a white line (length =1.125 dva, color: white) was presented and participants had 3 s to estimate the hidden target location for this trial and indicate their guess either by looking at it (pro-saccade) or by looking opposite of it (anti-saccade) and fixate their estimated location for 0.5 s. Thereafter, participants rated their level of confidence in their guess on a discrete scale from 1 to 6, where 1 means very uncertain and 6 means very certain about the target location. The confidence rating had to be done within 4 s by pressing the corresponding key on the keyboard (keys S, D, F, J, K, L corresponding to confidence level 1–6, respectively).

Participants were told that they had 20 trials to guess the location of the hidden target, after which a new hidden target had to be found. To estimate the hidden target location participants had to closely monitor the location of a line that was presented in every trial and served as a visual hint. The hidden target location was the mean of a von Mises distribution and each visual hint was a sample drawn from this distribution (Dekleva et al., 2016). Hence, by paying attention to the location of the hint across trials, participants were able to infer the underlying hidden target location. Participants indicated their estimates by looking either at where they thought the hidden target was located on the ring (pro-saccade), or at a location directly opposite to it (anti-saccade). Ten consecutive trials of a block of 20 trials required pro-saccade responses, and the other 10 anti-saccade responses. Importantly, the location of the hidden target in a block of 20 trials stayed the same and did not change after the switch in the required response type. The type of the required response (either pro-saccade or anti-saccade) was visually indicated by an instruction display presented every 10 trials. Participants were instructed to perform the same type of response for 10 trials in a row, until the response type changed.

In total, there were 40 hidden target blocks. The target location of each block, which is the mean of the von Mises distribution, was randomly drawn from a fixed set of 20 locations evenly distributed on the circle. Thus, each location only appeared twice during the experiment. To familiarize the participants with the connection between the hints and the hidden target location, 10 training trials were performed in the beginning of each experiment. After the 10 training trials, participants saw all the 10 lines together on the screen, as well as the correct hidden target location. Furthermore, after each hidden target block, participants saw where the actual hidden target location was, but they did not receive feedback about their performance on a trial-by-trial basis. As such, in our experiments, learning was unsupervised.

Four different experimental conditions, counter-balanced across blocks, were tested. Each block was either easy or hard, controlled by adapting the concentration of the von Mises distribution, and either ordered with first pro-saccade then anti-saccade, or first anti-saccade then pro-saccade response (Fig. 1*F*). For the easy task condition, the concentration of the von Mises distribution (defined by к which is a measure of dispersion, where 1/к is equivalent to the variance σ^2^_dist_ of the distribution) was 30 (σ_dist_ ∼10°), for the hard task condition it was 5 (σ_dist_∼26°) and for the training it was 80 (σ_dist_ ∼6°). As the concentrations of these distributions are relatively large, we could treat the von Mises distribution as a normal distribution and use standard statistics.

#### Calibration task

The aim of this task was to quantify the participant-specific motor error of the visually driven pro-saccades and anti-saccades. Each block of this task consisted of 20 trials, from which the first 10 were pro-saccades and the last 10 were anti-saccades. On each trial, one out of 10 equally distributed locations on the circle (same circle as in the hidden target task) were selected and a target line was presented at that location (Fig. 1*C*). Participants were instructed to look either directly at the displayed line (pro-saccade in the first 10 trials), or directly opposite to where it appeared (anti-saccades, second 10 trials). Additionally, it was highlighted that this task is completely independent of the hidden target task. Each trial consisted of an initial fixation phase, where participants had to fixate on the white cross in the middle of the screen for 0.5 s. After that, a white line appeared and participants had to either look at it or opposite of it (Fig. 1*D*). In the beginning of a block of 10 trials, participants received an instruction display indicating whether they had to perform pro-saccades or anti-saccades during the upcoming trials.

#### Successful response

For both tasks, a successful response was defined as follows. Participants had to move their gaze from the central fixation point toward a peripheral location on the ring. As soon as the gaze moved away from the fixation point by more than a radius of 5.375 dva, a successful response was possible. To complete the response, participants furthermore had to hold their gaze on their intended landing position for 0.5 s. During online measurements, as soon as the gaze crossed the 5.375-dva threshold, we calculated the mean and standard deviation of the gaze data in a moving window of 0.5 s. Whenever the standard deviation of the last 0.5 s of the gaze data fell below 1 dva, the gaze shift from the fixation point toward a peripheral location was considered as a successful response in that trial.

### Experiment 2

The second experiment served as a control that participants had indeed understood the task structure. Specifically, we aimed to ensure that participants were aware that the target location stayed the same across all 20 trials of a block of the hidden target task and did not change after a switch of response modality in the same block (i.e., after trial 10). To this end, we used the same experimental design and procedures as in experiment 1 with the exception of the following modifications: (1) we slightly modified the instruction slides during the experiment to enforce the notion that all 20 trials within one block belonged to the same hidden target location and did not change after a switch in response modality; and (2) we asked the participants after each block to report whether they were aware that all 20 trials belonged to the same hidden target location. They had to press a button to indicate their response, either yes or no.

### Data analysis

#### Data preprocessing

**Figure 2.**
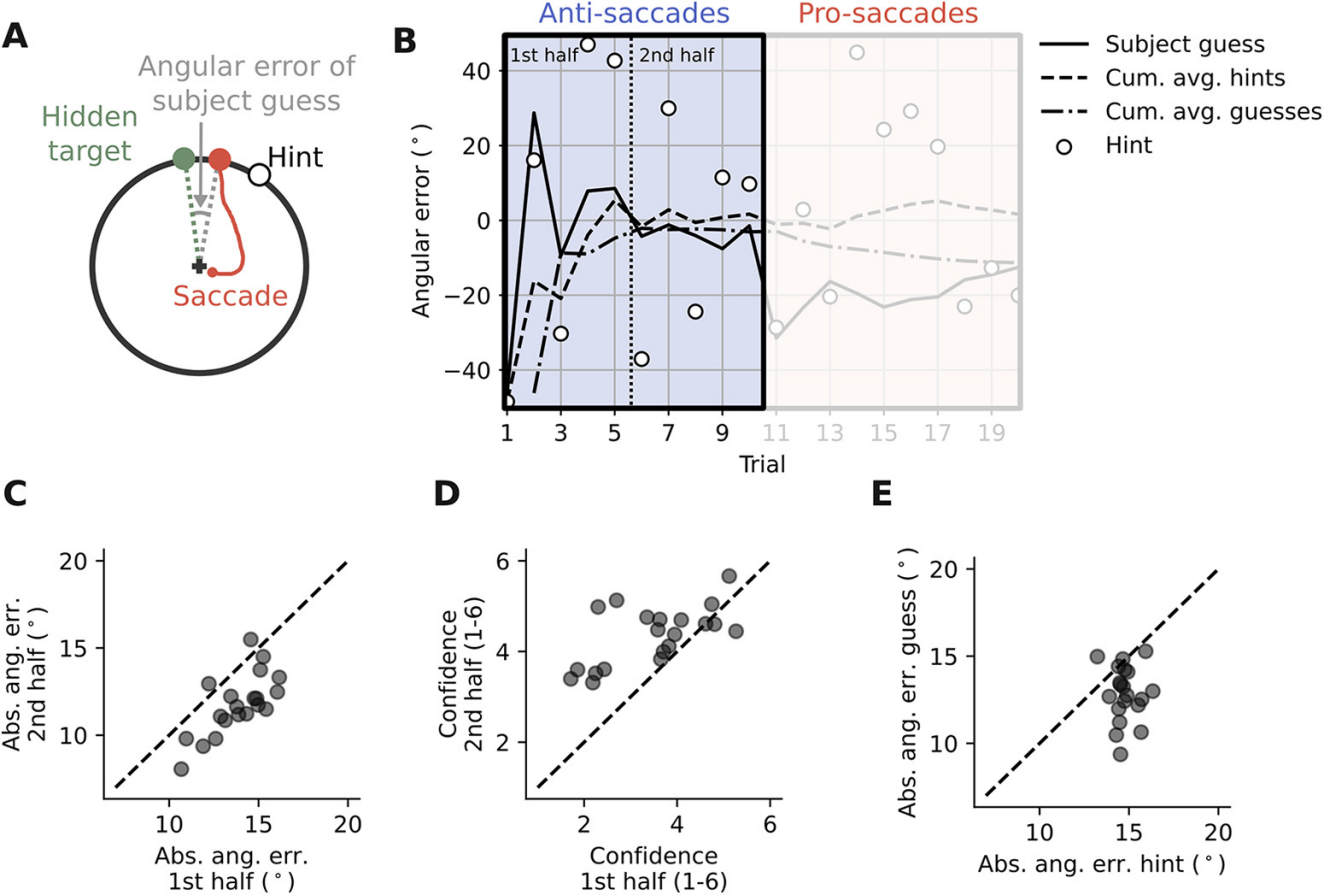
Participants successfully accumulate information and learn on a short time scale. ***A***, The angular difference between the participant’s guess and the true location of the hidden target was used to measure learning. ***B***, Example block. To test learning, we compared the performance in trials 1–5 (first half) to the performance in trials 6–10 (second half). ***C***, The absolute angular error in the second half is lower than in the first half (paired *t* test: *t* = 7.25, *p* < 0.0001, *N* = 20). ***D***, Participants’ confidence is higher in the second half than in the first half (paired *t* test: *t* = −4.39, *p* = 0.0003, *N* = 20). ***E***, The absolute angular error of participants is lower than the absolute angular error of the visual hints, i.e., participants’ guesses are closer to the center of the von Mises distribution compared with the presented visual hints (paired *t* test: *t* = 4.92, *p* = 0.0001, *N* = 20).

The recorded raw eye movement data were transformed to MATLAB files by using a MATLAB library for eye movement analysis (Manohar, 2019). Participants’ estimates in each trial were calculated offline by averaging the eye movement data of the last 100 ms, out of the total 500 ms necessary for a successful response. No further *post hoc* constraints on gaze data were used to identify successful responses beyond the method used online and described above (Successful response). The main eye movement parameter that we analyzed was the angular distance between the participant’s estimate and the true hidden target location (Fig. 2*A*). Failed trials were excluded from the analysis. A trial could fail because of three reasons. First, there could have been a disturbance with the eye tracking system or the participant’s calibration so that the gaze position was not correctly detected, which made it necessary to re-calibrate the eye tracker. Second, the participant could have been too slow to indicate their guess in time (3 s). Third, to exclude erroneous pro- instead of anti-saccades and vice versa, we analyzed the distribution of angular errors and found a bimodal distribution. We set a threshold at 100°, which separated both modes. Thus, every trial with an absolute angular error bigger than 100° was categorized as failed because of the wrong response type. Number of failed trials because of the first two reasons were 12/800 and 13/800 in pro and anti of the calibration task, and 150/4000 and 132/4000 in pro and anti of the hidden target task, respectively. Based on the third reason, 59/800 and 43/800 were marked as failed in pro and anti of the calibration task and 104/4000 and 196/4000 in pro and anti of the hidden target task. Hence, less than <9% of trials of each condition were excluded from further analysis.

#### Statistical analysis

**Figure 3.**
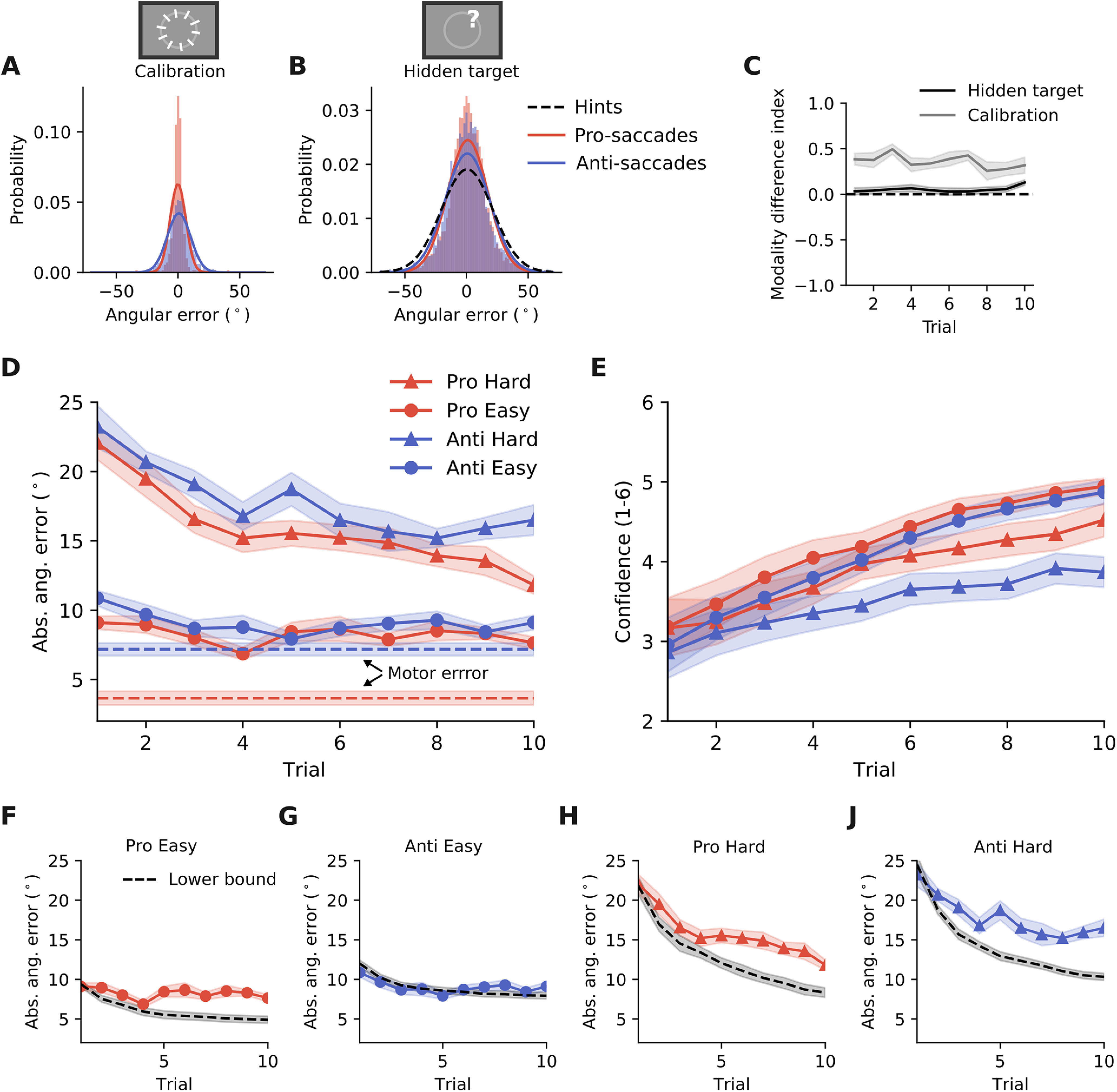
Similarity of learning curves across response modalities. ***A***, The distribution of the angular error for pro-saccade and anti-saccade response in the calibration task. ***B***, The distribution of the angular error for pro-saccade and anti-saccade response in the hidden target task. ***C***, The modality difference index quantifies the difference between the absolute angular error in pro-saccade and anti-saccade trials. The shaded area indicates the SEM. ***D***, Time course of the absolute angular error for each of the four different conditions (two response types × two difficulties). Here and in the following panels, except stated otherwise, shaded areas represent the SEM (*N* = 20). ***E***, Time course of the confidence ratings for each of the four different conditions. ***F–J***, Participants’ learning curves compared with the lower bound. The lower bound is given by taking the cumulative average of all hints presented so far and adding the error because of motoric noise, estimated from the calibration task.

Statistical analyses were done using R and Python. To fit the linear regression models, we either used the lm function in R or the OLS function from the python package statsmodels. To evaluate learning we used paired, two-sided *t* tests to compare several parameters within a participant. In these cases (Fig. 2), the assumption of normality was tested by applying a Shapiro–Wilk test to the data. We did not use circular statistics as subjects’ responses were highly localized on the ring (Fig. 3*A*,*B*). Learning was assessed by measuring the decrease in the absolute angular error between the participant’s estimate and the true hidden target location across trials.

**Figure 4.**
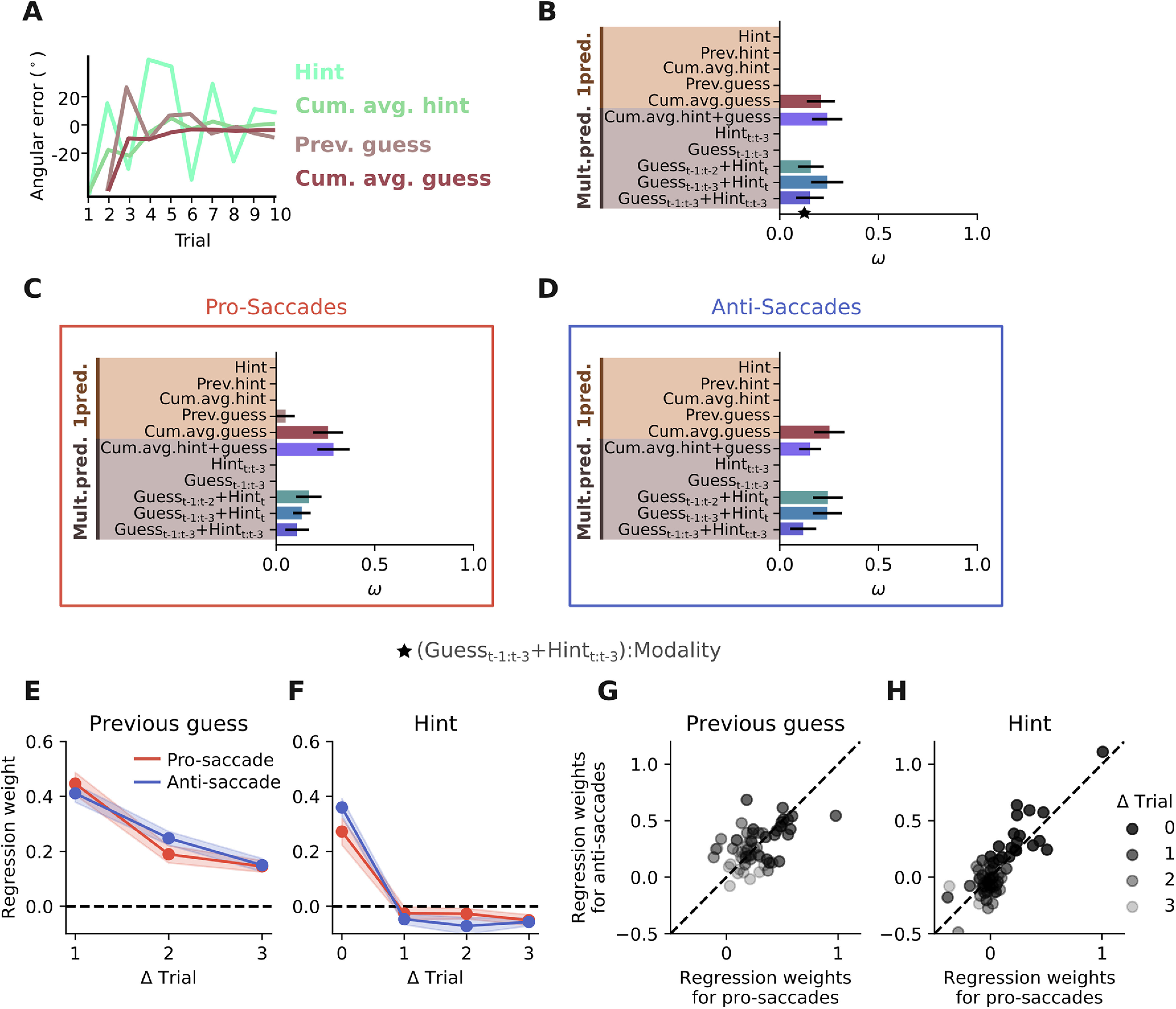
Learning strategy is similar across response modalities. ***A***, Different predictors used to explain the participants’ single trial estimates (i.e., angular error). ***B***, Model comparison between various single and multiple predictor models. Shown are the weights ω, which represent the probability that a model is the best among the ones considered. Error bars indicate SEM (*N* = 20). ***C***, ***D***, Same as ***B*** but performed on two different datasets, one consisting only of pro-saccade response trials (***C***), the other consisting only of anti-saccade response trials (***D***). ***E–H***, Regression weights for a model including participants’ last three guesses and the current and last three visual hints. Shaded area indicates SEM (*N* = 20). ***E***, Regression weights put on the last three guesses. ***F***, Regression weights put on the current, as well as the last three hints. ***G***, ***H***, Participants put similar weight on guesses and hints in pro-saccade and anti-saccade response trials (paired *t* test for previous guess: *t* = −0.38 *p* = 0.70; paired *t* test for hint: *t* = −0.26 *p* = 0.79). The weights put on previous hints and guesses were validated using separate models for hints and guesses (Extended Data Fig. 4-1). To select the relevant number of time steps in the past to include in the model comparison in B (see Table 1 for model definitions), we used a stepwise regression approach (Extended Data Fig. 4-2). Results shown in ***B*** were validated by varying the dataset used to fit the models (Extended Data Fig. 4-3), looking at best single subject models (Extended Data Fig. 4-4), and splitting the data according to task difficulty and response type (Extended Data Fig. 4-5).

**Figure 5.**
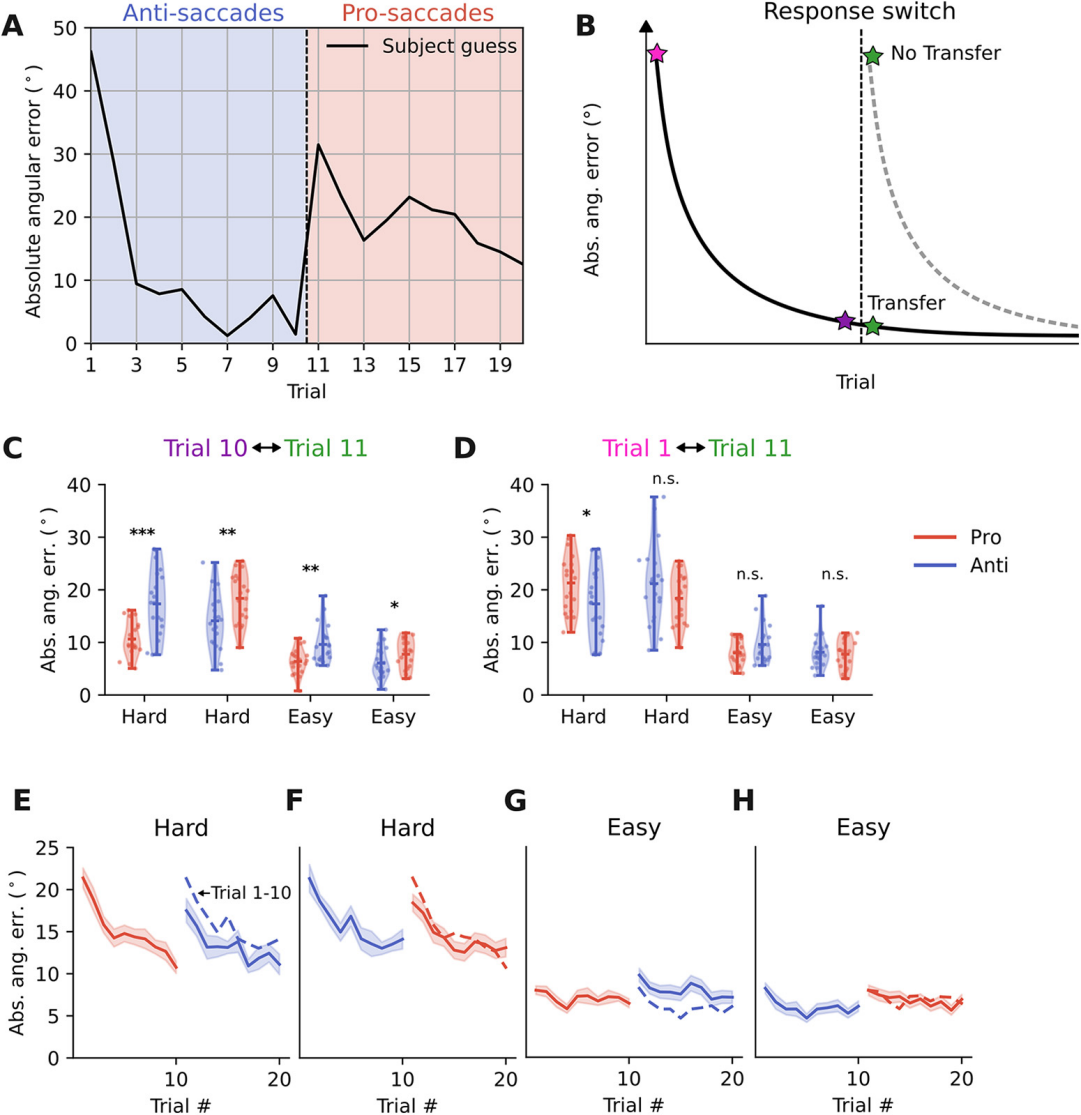
Drop in performance after response switch. ***A***, To test the knowledge transfer hypothesis, we analyzed all trials within a block, encompassing trials before and after the response switch. We specifically focused on the difference between trial 10 (before response switch) and trial 11 (after response switch). The same example block as in Figure 2 is shown. ***B***, If knowledge is transferred, we expect similar performance in trial 11 and trial 10. In contrast, if no knowledge is transferred, we expect similar performance in trial 11 and trial 1. To analyze the difference because of statistical learning only, we subtracted the motor error estimated from the calibration task to make pro-saccade and anti-saccade trials more comparable. ***C***, Comparison of performance in trial 10 and 11 for the four different experimental conditions (difficulty × pro/anti order). Each dot represents one subject and horizontal bars indicate mean and extreme values. ***D***, Same as ***C*** but for comparison between trial 1 and trial 11; ***p < 0.001, **p < 0.01, *p < 0.05, n.s. *p* > 0.05. ***E–H***, Performance time course for different difficulty levels and pro-/anti-saccade orders. Dashed colored lines represent performance in trials 1–10. Shaded area indicates the SEM (*N* = 20). The results for performance were corroborated by analyzing confidence levels, which showed a similar drop from trial 10 to trial 11 (Extended Data Fig. 5-1, Fig. 5-2). We tested whether the drop in performance was related to the fact that subjects might have misunderstood the task. A control experiment (see Materials and Methods, experiment 2) confirmed that although subjects were aware that all 20 trials belonged to the same hidden target location, they were not able to integrate information across the response switch (Extended Data Fig. 5-3). Furthermore, we validated that the subtraction of the motor error is plausible and that there is no temporarily increased motor error after the response switch (Extended Data Fig. 5-4).

**Figure 6:**
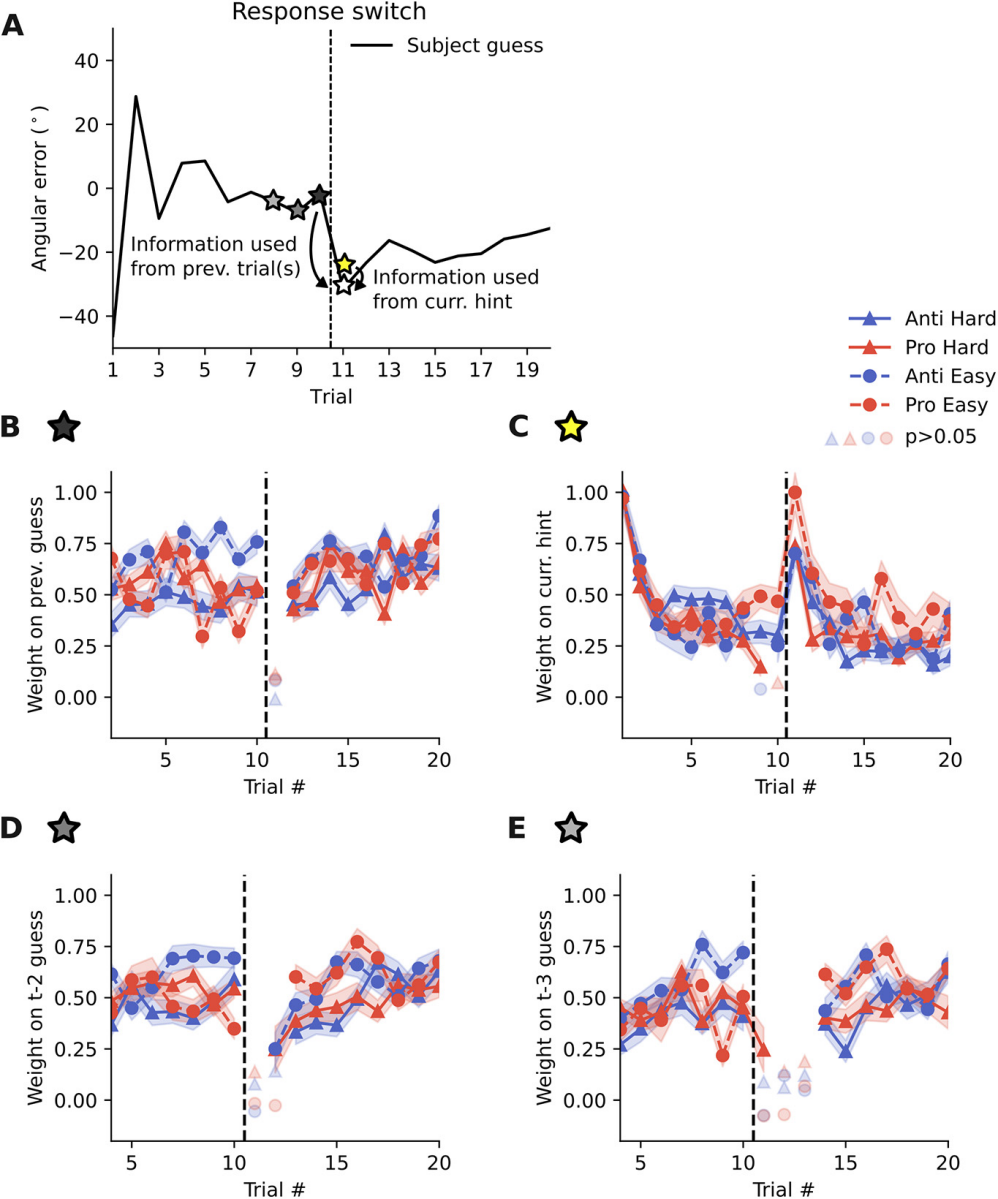
Almost no knowledge transfer between visuo-motor modalities. ***A***, To test whether there is any knowledge transferred from the experience with one response type to the other, we regressed the current guess against previous guesses/current hint. ***B***, Participants’ estimates at trial 11 (after response switch) are independent of the estimates at trials 10 (before response switch). In contrast, at every other time point, participants use previous experience to inform their current guess. Shaded area here and in the remaining panels indicates the SEM (*N* = 20). ***C***, At trial 11, participants highly rely on the information coming from the current hint. ***D***, ***E***, Similar to the lack of transfer from one trial to the next (***B***), there is also no transfer from trials further in the past across the response switch (trials 11–12 for t-2; trials 11–13 for t-3). In ***B***, ***D***, ***E***, non-significant regression weights with a *p* > 0.05 are shown in opaque.

To compare the performance difference between pro-saccades and anti-saccades, we calculated a “modality difference index” (Fig. 3*C*). For this, we first calculated the mean of the absolute angular error for pro-saccade and anti-saccade response trials. The modality difference index is then given by the difference between the two means, divided by their sum (calculated per subject). We started our analysis by only using the data from the first 10 trials of each block (Figs. 1-4). Only when the transfer between modalities was examined, we used all 20 trials of each block (Figs. 5, 6).

10.1523/ENEURO.0032-21.2021.f4-1 Extended Data Figure 4-1 Memory traces for previous guesses and hints. This figure is supplementary to Figure 4 in the main text. Here, we quantified how much weight is put on previous hints or guesses, tested in separate models each including either only the guesses or only the visual hints. Data were pooled across all experimental conditions, so from pro-saccade and anti-saccade, as well as from easy and hard task condition. ***A***, Regression weights of the model including previous guesses as a predictor. ***B***, Same as ***A*** for a separate model including visual hints as a predictor. ***C***, Comparison of the model shown in ***A*** against the model shown in ***B***. It can be seen that the previous guess models consistently outperformed the visual information/hints models, confirming the finding that subjects’ behavior is better predicted by previous actions than external visual information. ***D***, ***E***, Same as ***A***, ***B***, but showing the single subject regression weights instead of the average weights. Download Figure 4-1, TIF file.

10.1523/ENEURO.0032-21.2021.f4-2 Extended Data Figure 4-2 Stepwise regression to select relevant predictors for model comparison. This figure is supplementary to Figure 4 in the main text. Here, we used stepwise linear regression (train function with method “leapForward” from the Caret package in R) to estimate the relevant number of past trials that should be included in our main model comparison analyses (Fig. 4; Table 1). We tried to predict the angular error in trial 10 from either all previous guesses (***A***), all observed visual hints (***B***), or a combination of both (***C***, ***D***). Shown is the cross-validated fit accuracy (10-fold), measured as the root-mean-squared error (RMSE). Thereby, we found for each individual subject the number of trials (=predictors) that were included in the best fitting model (red dot), indicated by the lowest RMSE. To identify not only the number, but also the type of information that best predicted performance in trial 10, we analyzed which timesteps of previous guesses and current and previous hints were included in the best combined model (***C***) and created a histogram indicating for how many subjects the specific predictor (either guess: G or hint: H) was included in the best model (***D***). Using this approach, we found that for most subjects a model with six or less predictors, including a combination of previous guesses and visual hints, is best in predicting the angular error of the current trial. Thereby, we focused our main analysis (Fig. 4; Table 1) on three timesteps in the past (up to t-3). This allowed us to predict not only the behavior in trial 10, as done here, but also the behavior from trial 4 to trial 10. A general trend in the main analysis, which is also apparent here, is that previous guesses are better in predicting current behavior, compared to current or previous visual hints [lower errors for models including guesses (***A***) compared to hints (***B***)]. Download Figure 4-2, TIF file.

10.1523/ENEURO.0032-21.2021.f4-3 Extended Data Figure 4-3 Model comparison results of single predictor models tested on trials 2–10. This figure is supplementary to Figure 4*B* in the main text. Our main modeling results were based on models that included up to three timesteps in the past (*n* = 3), where *n* was determined based on a model search approach (Extended Data Fig. 4-2). To this end, we included trials 4–10, allowing us to test the models on a consistent dataset. To test whether our results hold when we include earlier trials in a block, we repeated our analysis focusing only on single predictor models (Table 1, models 1–5), which allowed us to use the data from trial 2 to trial 10. Similar to our main results, the best single predictor model was the one based on the cumulative average of past guesses. Download Figure 4-3, TIF file.

10.1523/ENEURO.0032-21.2021.f4-4 Extended Data Figure 4-4 Model comparison results for single subjects. This figure is supplementary to Figure 4*B* in the main text. Here, we show the model comparison results for each single subject individually. Download Figure 4-4, TIF file.

10.1523/ENEURO.0032-21.2021.f4-5 Extended Data Figure 4-5 Dependency of model comparison results on experimental condition. This figure is supplementary to Figure 4 in the main text. ***A–D***, Model comparison results for data split according to pro-/anti-saccades and easy/hard task difficulty. Overall model comparison results did not depend on the response type or task difficulty. ***E***, Example model prediction for a held-out data block. Data were pooled across experimental conditions, identical to the procedure used in Figure 4. ***F***, Summary of model performance on held-out data, calculated using 10-fold cross-validation and the Caret package in R. Data were pooled across experimental conditions, as in Figure 4. Model performance was measured as the root-mean-squared-error (RMSE) for the difference between the true and the predicted angular error in the held-out data. The null model (also in the Caret package) corresponds to not using any predictor, but only fitting the intercept. Download Figure 4-5, TIF file.

**Table 1:** Overview of the models used to assess sensorimotor statistical learning

	Name	Equation
1	Hint	$Guess_{t}=\beta_{0}+\beta_{1}Hint_{t}$
2	Prev. hint	$Guess_{t}=\beta_{0}+\beta_{1}Hint_{t-1}$
3	Cum. avg. hint	$Guess_{t}=\beta_{0}+\beta_{1}CumAvgHint_{t}$
4	Prev. guess	$Guess_{t}=\beta_{0}+\beta_{1}Guess_{t-1}$
5	Cum. avg. guess	$Guess_{t}=\beta_{0}+\beta_{1}CumAvgGuess_{t}$
6	Cum. avg. hint+guess	$Guess_{t}=\beta_{0}+\beta_{1}CumAvgHint_{t}+\beta_{2}CumAvgGuess_{t}$
7	Hint_t:t-3_	$Guess_{t}=\beta_{0}+\beta_{1}Hint_{t}+\beta_{2}Hint_{t-1}+\beta_{3}Hint_{t-2}+\beta_{4}Hint_{t-3}$
8	Guess_t-1:t-3_	$Guess_{t}=\beta_{0}+\beta_{1}Guess_{t-1}+\beta_{2}Guess_{t-2}+\beta_{3}Guess_{t-3}$
9	Guess_t-1:t-2_+Hint_t_	$Guess_{t}=\beta_{0}+\beta_{1}Guess_{t-1}+\beta_{2}Guess_{t-2}+\beta_{3}Hint_{t}$
10	Guess_t-1:t-3_+Hint_t_	$Guess_{t}=\beta_{0}+\beta_{1}Guess_{t-1}+\beta_{2}Guess_{t-2}+\beta_{3}Guess_{t-3}+\beta_{4}Hint_{t}$
11	Guess_t-1:t-3_+Hint_t:t-3_	$Guess_{t}=\beta_{0}+\beta_{1}Guess_{t-1}+\beta_{2}Guess_{t-2}+\beta_{3}Guess_{t-3}+\beta_{4}Hint_{t}+\beta_{5}Hint_{t-1}+\beta_{6}Hint_{t-2}+\beta_{7}Hint_{t-3}$

10.1523/ENEURO.0032-21.2021.f5-1 Extended Data Figure 5-1 Time course of confidence rating. Download Figure 5-1, TIF file.

10.1523/ENEURO.0032-21.2021.f5-2 Extended Data Figure 5-2 Confidence drops after response switch. Figures 5-1 and Figures 5-2 are supplementary to Figure 5 in the main text. The confidence ratings demonstrated the same results as observed by analyzing the absolute angular errors of the eye movements, as there was a decrement in confidence from trial 10 to trial 11 (as shown in Extended Data Fig. 5-1*A* for experiment 1 as well as in Extended Data Fig. 5-1*B* for experiment 2), in all experimental conditions. The drop in confidence between trial 10 and trial 11 was significant (Extended Data Fig. 5-2*A*; paired *t* test; *t* = 3.67, *p* = 0.0016, *N* = 20). The confidence of trial 11 was not different from trial 1 (Extended Data Fig. 5-2*B*; paired *t* test; *t* = –1.78, *p* = 0.0906, *N* = 20). These results support the observation that after a switch in response modality, learning starts from an almost naive level. Download Figure 5-2, TIF file.

10.1523/ENEURO.0032-21.2021.f5-3 Extended Data Figure 5-3 Second experiment with reinforced instructions shows similar results. This figure is supplementary to Figures 5, 6 in the main text. These results demonstrate that we obtained similar results when participants were explicitly instructed that the location of the hidden target remained the same after a switch. Additionally, participants had to report whether they were aware of this rule, thus reinforcing the instructions. ***A***, Also, in this experiment, performance dropped to almost naive levels after a switch in response type. ***B***, The majority of participants reported to be aware of the rule. ***C***, The weighting of previous guesses dropped between trial 10 and trial 11 (when the switch occurred), and instead (***D***), more weight was put on visual hints. Download Figure 5-3, TIF file.

10.1523/ENEURO.0032-21.2021.f5-4 Extended Data Figure 5-4 Motor error estimation. This figure is supplementary to Figures 3, 5 in the main text. Here, we tested whether our assumption that participants’ estimation error in the hidden target task calculated as a combination of two independent sources of uncertainty, i.e., the motor noise (estimated from the calibration task) and statistical uncertainty (estimated from the distribution of visual hints in the hidden target task), was plausible. ***A***, Time course of motor error in the calibration task. ***B***, In the following, we examined trial 1 of the hidden target task and compared subjects’ actual performance to the theoretical prediction of adding motor noise and statistical uncertainty. If our assumption that both noise sources are independent and thus can be added is plausible, we would expect that the actual (*x*-axis) and predicted (*y*-axis) data would match. ***C–F***, Results for all four experimental conditions. Each dot represents one subject (*N* = 20). Paired *t* tests were performed to test whether there is a significant difference between the theoretical prediction and the actual data and results are shown in the respective panels. Download Figure 5-4, TIF file.

#### Theoretical bounds for learning performance

To evaluate participants’ performance, we computed the theoretical lower bounds on the absolute angular error they could potentially achieve by using all the information that was available to them. Participants could use the previously seen visual hints and the memory of their previous motor actions (referred to as previous guesses) to infer the most probable location of the target on each trial. These sources of information are error prone since on each trial participants had only seen a limited number of visual hints (i.e., sampling error) and their previous responses contained motoric noise. We assumed that these two sources of error are independent. The error because of the limited number of visual hints was calculated as the cumulative mean of all hints seen so far, which represents an optimal way of combining samples over time to estimate the mean of a distribution. A constant motor error, measured for each participant during the calibration task, was used to represent the motoric noise. The variance of the joint estimate, derived from combining visual hints and motor actions, was then calculated by adding the variance of the two sources, based on the assumption of their independence. To understand how participants used different sources of information to make decisions on a trial-by-trial basis, we employed a detailed model-comparison approach as described below.

#### Modeling participants’ behavior

We used linear regression models (lm package in R) to analyze the single subject behavior. To this end, the angular error of participants’ estimate in a given trial (i.e., their guess in trial t, *Guess_t_*) was fitted by models having a diverse set of independent variables as shown in Table 1.

The five single predictor models we tested were (1) using the visual hint in each trial; (2) using the visual hint in the preceding trial (t-1); (3) using the cumulative average of all visual hints so far; (4) using the guess from the previous trial; and (5) using the cumulative average of all previous guesses (Fig. 4*A*; Table 1). The dependent variable was the angular error of a participant’s estimate in a certain trial (*Guess_t_*). The independent variable was the angular error of the estimate, given by one of the above-mentioned strategies. In a second stage, we tested linear regression models with multiple independent variables. For this, we included previous guesses from up to three time steps in the past, as well as the visual hints from the current and up to three time steps in the past. Our choice of including three trials in the past was inspired by a naive model search approach (Extended Data Fig. 4-2), which indicated that including up to three trials in the past provided the best fit to the participants’ data. Thus, in our multipredictor models only the data from trial four to trial 10 was included, allowing all models to be tested on the same data. Each of the described regression models was fitted to the single subject data using R’s lm package. To compare the models, we calculated BIC, δ BIC, and Bayesian weights (Burnham and Anderson, 2004), to assess the likelihood of each model being the best fit to the data (cf below, Evaluating model performance). Since these values are normalized, they can be used to determine the model that on average best fits the participants’ data.

#### Evaluating model performance

To compare the different linear regression models presented above we used a model comparison evaluation based on Bayesian weights (Burnham and Anderson, 2004). For this, we first calculated BIC values for each model. Each BIC value was rescaled by calculating ΔBCI, which is calculating the difference to the smallest BIC value in the group of models considered. This forces the best model to have ΔBCI = 0 and the other models to have positive values. We then calculated Bayesian weights ω, as described in (Burnham and Anderson, 2004). The Bayesian weights of all tested models in Figure 4*B* sum up to 1 and define the probability of being the best model, among the one tested.

## Results

To investigate the dynamics of sensorimotor statistical learning of a spatial prior and its dependence on the response modality, we designed an experiment where participants had to find a hidden target and indicate their guess by either a pro-saccade or an anti-saccade. Participants learned the location of each hidden target within 20 trials, of which a block of 10 trials required pro-saccades and the other block of 10 trials required anti-saccades as the response modality (Fig. 1*A*). This design allowed us to probe whether the learning dynamics shows dependencies on the response modality, hence being modality specific. We also used another task, referred to as the calibration task, to estimate each participant’s motoric noise during the visually driven execution of pro-saccades or anti-saccades. In contrast to the calibration task, in the hidden target task the main error source is the uncertainty regarding the hidden target location. As the same motor system is used in both the hidden target task as well as the calibration task, we assumed that the motor noise affecting participants’ performance in both tasks is equal. As the motor noise in the calibration task is not time-dependent, we assumed that all time-dependent performance improvement during the hidden target task reflected statistical learning, defined as the reduction in the uncertainty regarding the location of the hidden target.

### Participants successfully accumulate information and learn on a short time scale

To establish that participants were in general able to learn on a short timescale, we initially focused on the first 10 trials of each hidden target block (Fig. 2*B*). In this case, in all 10 trials participants responded by using the same modality, either exclusively by pro-saccades, or exclusively by anti-saccades. To quantify performance, we calculated the absolute angular error between the saccade endpoint and the hidden target location (Fig. 2*A*). By comparing the average absolute angular error in the first five trials with the average absolute angular error in the last five trials, we found that most of the participants were able to improve their estimates of the target location (i.e., their guesses) during this short timescale (paired *t* test: *t* = 7.25, *p* < 0.0001, *N* = 20; Fig. 2*C*). In line with performance, participants’ confidence about the accuracy of their guesses increased during the 10 trials (paired *t* test: *t* = −4.39, *p* = 0.0003, *N* = 20; Fig. 2*D*). Additionally, the absolute angular error of participants’ guesses was lower than the absolute angular error of the visual hints, meaning that participants’ guesses were closer to the center of the von Mises distribution compared with the presented visual hints (paired *t* test: *t* = 4.92, *p* = 0.0001, *N* = 20; Fig. 2*E*). This shows that participants were able to combine information across trials and thereby improve their estimates of the target location, rather than just following the current visual hint. Hence, we can conclude that participants showed some form of statistical learning during the first 10 trials of the hidden target task.

### Similarity of learning curves across response modalities

To work out whether statistical learning is similar across response modalities, we contrasted the learning performance in pro-saccade versus anti-saccade trials in the hidden target task, as well as in the calibration task. First, we calculated the mean and the standard deviation of the respective angular error distributions, pooled across participants (Fig. 3*A*,*B*; Table 2), as well as the performance measure used here and in the following, the absolute angular error (Table 2).

**Table 2:** Angular error distribution for the calibration and the hidden target task

Task	Modality	Mean	SD	Absolute angular error
Calibration	Pro-saccade	−0.1°	6.3°	3.5°
Calibration	Anti-saccade	0.7°	9.5°	7.1°
Hidden target	Pro-saccade	1.1°	16.9°	12.1°
Hidden target	Anti-saccade	0.1°	18.4°	13.4°
Hidden target	Hints	0.1°	20.6°	14.7°

Mean and SD for distributions shown in Figure 3*A,B*. The rightmost column indicates the average absolute angular error in each task condition, estimated from the subjects’ guess, or, for the last row, from the distribution of visual hints.

For both modalities and both task difficulties, the absolute angular error in the hidden target task was higher than in the calibration task (pro hard: *t* = 13.8, *p* < 0.0001; pro easy: *t* = 8.8, *p* < 0.0001; anti hard: *t* = 14.1, *p* < 0.0001; anti easy: *t* = 2.9, *p* = 0.0084; paired *t* test each with *N* = 20). This makes sense as in the former task the target location was uncertain whereas in the latter it was not. Furthermore, the performance in pro-saccade and anti-saccade blocks was only significantly different during the calibration task, but not during the hidden target task (calibration task: *t* = 5.6, *p* < 0.0001; hidden target task easy: *t* = 1.7, *p* = 0.1106; hidden target task hard: *t* = 1.8, *p* = 0.0903; paired *t* test each with *N* = 20). To quantify this difference further, we calculated a modality difference index (described in Materials and Methods), which was consistently higher in the calibration task compared with the hidden target task (Fig. 3*C*). These results provide first evidence that statistical learning is similar for pro-saccade and anti-saccade response.

So far, we have shown that the average performance in the first 10 trials does not seem to depend on the modality used for indication, pro-saccades or anti-saccades. Nevertheless, the specific time course of statistical learning could still be modality dependent. For this, we next looked at the performance in a trial-dependent manner (Fig. 3*D*). As expected from the analysis in Figure 2*C*, we observed that the performance is slowly increasing with each trial, indicating that the participants were using the information given in each trial to improve their estimate of the hidden target location. This general behavior was also reflected in the time course of subjects’ own confidence (Fig. 3*E*). In line with the previous, trial-independent analysis, we found that the performance for pro-saccades and anti-saccades was very similar (Fig. 3*D*, compare red and blue). This similarity seemed especially interesting when compared with the observed difference in motor error during the calibration task (Fig. 3*D*, dashed line). In summary, inspection of the learning curves of pro-saccade and anti-saccade indication supported our initial results suggesting that statistical learning is independent of the response modality.

### Performance is mostly suboptimal

Since at each point in time, participants have only seen a limited number of samples from the underlying distribution of the target location (in form of visual hints), it is impossible to correctly estimate the distribution’s mean, i.e., the hidden target location in our task. In principle, only for an infinite number of samples, the estimated mean equals the true distribution mean. For the hidden target task, this means that participants could only reach zero absolute angular error if an infinite number of trials were seen. Taking this into account, we can compare participants’ performance to the time course of the theoretical statistical uncertainty because of seeing a limited number of samples (i.e., sampling error). This provides a lower bound γ on participants’ performance:

(1) $$\gamma =\sqrt{\gamma_{Motor}^{2}+\gamma_{Hint}^{2}},$$

where $\gamma_{Motor}$ is the motor error measured in the calibration task and $\gamma_{Hint}$ is the absolute angular error related to seeing only a limited number of samples (the visual hints). Comparing participants’ performance to this lower bound showed that for the first trial in each hidden target block, participants performed as well as they possibly can (Fig. 3*F–J*; Extended Data Fig. 5-4), independent of the task difficulty or the used response modality, pro-saccades or anti-saccades. The fact that the estimated lower bound matched subject performance in the first trial, where no learning has yet happened, also supports the plausibility of the assumption that the motor noise and the statistical noise can be added to derive an estimate of participants’ performance (also see Extended Data Fig. 5-4). However, beyond the first trial and as soon as subjects needed to combine multiple samples to estimate the hidden target location, they performed suboptimally in most conditions, as can be seen in the difference of their estimates to the theoretical lower bound (Fig. 3*F–J*, black dashed line is optimal).

Interestingly, we found minor differences in terms of performance between the four different conditions we tested (pro/anti × hard/easy). Three out of four conditions showed clear suboptimal behavior (Fig. 3*F*,*H*,*J*). However, in the condition where the visual hint distribution was more concentrated (i.e., the easy condition) and anti-saccades were used as the response modality, performance was close to optimal (Fig. 3*G*). As described before, this finding cannot be ascribed to the fact that our estimate of the motor error was flawed, thereby artificially improving the measured performance, as the estimate for Trial 1 closely matched what we observed in participants’ behavior (Extended Data Fig. 5-4). We suspect that in this condition, performing an anti-saccade discourages a bias to follow the visually presented lines too much, and hence can be beneficial in producing optimal performance. Nevertheless, the differential learning of pro-saccade and anti-saccade responses with respect to an optimal lower bound on performance was not present in the hard task condition (Fig. 3*H–J*), indicating that the optimal learning for anti-saccades only occurred when the visual hints where narrowly distributed.

### Learning strategy is similar across response modalities

So far, we have seen that statistical learning in the pro-saccade and in the anti-saccade context is similar in terms of the absolute performance and the shape of the learning curve. Next, we wanted to test whether participants used different learning strategies in the pro-saccade and anti-saccade blocks. The first candidate strategy would be to only look at the visual hints in each trial, which would mean that no learning is happening, and that participants’ behavior is only visually driven. Instead, the optimal strategy, which would result in the lower bound we calculated before, would be to calculate the cumulative average of every hint seen so far in each trial. Besides considering the hints, visually presented to the participants, we can also imagine that the behavior is driven by an internal state that promotes looking close to where one has been looking before, i.e., to follow previous guesses (Fig. 4*A*). To test these different hypotheses, we fit several single predictor linear regression models corresponding to each strategy (see Table 1). Through a model comparison (see Materials and Methods), we found that the best single predictor model is the cumulative average of previous guesses (Fig. 4B; Extended Data Figs. 4-3, 4-4). Splitting the data into pro-saccade and anti-saccade blocks and repeating the analysis showed that the best single predictor model does not depend on the used response type (Fig. 4*C*,*D*). Thus, this provides another piece of evidence that statistical learning in the presented task is independent of the response type.

In a second step, we tested several multipredictor models to get a more detailed view on participants’ learning strategy. To determine the number of trials in the past potentially having an influence on the current decision, we conducted a stepwise linear regression analysis, where all previous guesses and all presented hints thus far were included to estimate participant’s angular error in trial 10 (Extended Data Fig. 4-2). This showed that for most participants, models with a low number of predictors performed as well as or better than models including all past trials (Extended Data Fig. 4-2*A–C*). Furthermore, we found that the predictors most often included in the best model were the current visual hint and the three previous guesses (Extended Data Fig. 4-2*D*). Based on these results, we chose to include information from up to three timesteps in the past and specifically compared five different options of how previous guesses and presented visual hints can be combined (Table 1). The model comparison revealed that multipredictor models which relied on combinations of external and internal information, i.e., visual hints and previous guesses, performed much better than models that were based on only one of the two sources of information (i.e., either the previous three guesses or the current and previous three visual hints; Fig. 4*B*). This was consistent across subjects (Extended Data Fig. 4-4). Again, splitting the data into pro-saccade and anti-saccade blocks did not affect the main trend (Fig. 4*C*,*D*), nor did splitting the data into hard and easy task difficulty (Extended Data Fig. 4-5).

Lastly, to find the exact weighting participants put on their previous guesses and the current and previous hints, depending on the used response type, we fitted a model with all three previous guesses, the current and three previous hints, and the response type as predictors. We found that participants used external information only from the current trial, ignoring the hints from previous trials (Fig. 4*F*). Instead, to combine information across trials they relied on their internal estimations from the past (Fig. 4*E*). We obtained similar results when models that only included either the past guesses or the past visual hints were tested (Extended Data Fig. 4-1), excluding the possibility that our results were biased by the inherent covariation of previous guesses and previous visual hints. Including the response type in the model allowed to estimate separate regression weights for pro-saccades and anti-saccades. Again, we did not find any significant difference in the weighting participants put on their own guesses versus given hints, depending on whether they use pro-saccades or anti-saccades for response (Fig. 4*E–H*; paired *t* test for previous guess: *t* = −0.38 *p* = 0.70; paired *t* test for hint: *t* = −0.26 *p* = 0.79). In summary, we found that participants used a very similar learning strategy to solve the task, regardless of the response type.

### Drop in performance after response switch

Despite the similarity in learning performance and strategy, hinting at a general algorithm used for statistical learning in our task, it is still possible that learning happens for each visuo-motor modality in a very specific manner and that both just look similar in terms of performance and strategy. In this case, it would not be possible to generalize between modalities. To test this, we included trial 11 until trial 20 where participants had to continue looking for the same hidden target (and were also instructed that all 20 trials belong to one hidden target), but had to use the other visuo-motor modality than in trial 1 until trial 10 (Fig. 5*A*). We considered two alternative hypotheses. The first states that participants learn in a modality-independent fashion and store the acquired knowledge in an abstract form. This would allow for a complete transfer of previous experience to the new response modality (Fig. 5*B*, black solid line). The second hypothesis would be that participants perform trial 11 until trial 20 as if there was no previous experience, which would suggest a response modality-specific representation of the learned knowledge (Fig. 5*B*, gray dashed line). To test which of the two hypotheses better matched the data, we compared the performance in trial 10 (last trial before response switch) to the performance in trial 11 (first trial after response switch; Fig. 5*C*). Since trials before and after the response switch were performed using different response modalities and since pro-saccades and anti-saccades are associated with different motor errors (Fig. 2), we subtracted the respective motor error estimated from the calibration task from participants’ absolute angular errors during the hidden target task. This procedure was validated by demonstrating that the resulting estimate closely matched the ground truth statistical error, which in trial 1 is simply the uncertainty related to the spread of the visual hint distribution (Extended Data Fig. 5-4*C–F*). A repeated measures three-way ANOVA was performed on participants’ absolute angular error as the dependent factor and task difficulty, trial number (10 or 11), and pro-/anti-saccade order as independent factors. We found significant main effects of difficulty (*p* < 0.0001) and trial number (*p* < 0.0001), but the main effect of pro-/anti-saccade order was not significant (*p* = 0.291). There were also significant interaction effects between pro-/anti-saccade order and difficulty (*p* = 0.004), as well as between trial number and difficulty (*p* = 0.006). *Post hoc* paired *t* tests showed that the performance in trial 11 was overall worse than in trial 10, with a significant drop in performance in all four conditions (trial 11–trial 10 for pro-saccade first/hard: *t* = 4.05, *p* = 0.0007; for anti-saccade first/hard: *t* = 3.17, *p* = 0.005; for pro-saccade first/easy: *t* = 3.01, *p* = 0.007; for anti-saccade first/easy: *t* = 2.20, *p* = 0.04; compare Fig. 5*C*,*E–H*). This showed that there is an overall drop in performance after the response switch suggesting that the knowledge acquired during the first 10 trials is not fully transferred to trials after a response switch. The drop in performance is not likely to be because of a temporarily increased motor error after a switch in response type, as we did not observe a similar pattern during the calibration task (paired *t* test to compare absolute angular error in trial 20 and trial 11 of the calibration task: *t* = −1.5, *p* = 0.15; Extended Data Fig. 5-4*A*). These results were corroborated by analyzing confidence ratings, as we also observed a drop in confidence at trial 11 compared with trial 10, in line with our results on performance (paired *t* test trial 11–trial 10, *t* = 3.7, *p* = 0.0016; Extended Data Figs. 5-1, 5-2). Together, these results suggest that although the learning curves and strategies for pro-saccade and anti-saccade responses were highly similar (Figs. 3, 4), there was no full knowledge transfer from one response type to the other.

To test whether there is at least a minor improvement in performance related to experiencing 10 trials of the opposite response type, we compared the performance in trial 11 (after the response switch) with the naive performance in trial 1 (Fig. 5*D*). We used the same ANOVA analysis as before, only replacing trial 10 with trial 1. There was a statistically significant effect of difficulty (*p* < 0.0001), but not trial number (*p* = 0.103), or pro-/anti-saccade order (*p* = 0.789). There also was a significant interaction effect between difficulty and trial number (*p* = 0.003). *Post hoc* paired *t* tests showed that there was a significant improvement in performance from trial 1 to trial 11 only in the hard task condition when pro-saccade response was followed by anti-saccade response (trial 11- trial 1, *t* = −2.28, *p* = 0.034), but not in any other of the remaining three conditions (hard/anti-first: *p* = 0.207; easy/pro-first: *p* = 0.168; easy/anti-first: *p* = 0.679). Together, these results suggest that there is only very limited knowledge transfer from one response condition to the other, as performance was slightly increased compared with naive levels only in one out of four conditions (hard/pro-first).

As comparing performance before and after the modality switch necessarily includes the factor that pro-saccades and anti-saccades have inherently different performance because of motor noise (compare Fig. 2*A*,*B*), we also compared the performance within the same modality, either pro-saccade or anti-saccade response, between trial 1 and trial 11. To analyze whether performance overall improved in trial 11, compared with 1, we performed a similar repeated measures three-way ANOVA with modality, difficulty, and trial number as independent factors. Here, we did not subtract the motor error, since the effect of trial number was compared within, and not across response modalities, as previously done. We found a significant main effect of difficulty (*p* < 0.0001), but not modality or trial number, similar to our previous analysis subtracting the motor error. Additionally, we found a significant interaction effect between difficulty and trial number (*p* = 0.0020). *Post hoc t* tests confirmed that in only one out of the four experimental conditions there was a statistically significant improvement between trial 1 and trial 11 (trial 11 – trial 10 hard/pro: *t* = −2.2, *p* = 0.041; hard/anti: *t* = −1.75, *p* = 0.097; easy/pro: *t* = −0.2, *p* = 0.837; easy/anti: *t* = 1.9, *p* = 0.072). In summary, this analysis confirmed the results we obtained when comparing pro-saccades and anti-saccades directly, thus ruling out the possibility that our results are merely an artifact of subtracting the motor error measured in the calibration task.

### Almost no knowledge transfer between visuo-motor modalities

As modeling showed that participants relied on their previous guesses rather than the previously seen visual hints (Fig. 4*E*,*F*), we wished to test whether this is also the case across the time that a response switch occurs. For this, we calculated the regression weights on previous guesses in a time-dependent manner (Fig. 6*A*). If knowledge is not transferred between different visuo-motor modalities we would predict: (1) participants highly rely on the visual hint in trial 11 and do not use previous guesses to inform their decision; (2) they are also not able to use previous guesses further in the past, if these guesses were obtained before the response switch. Our analysis confirmed these two predictions. We found that participants did not use their previous guess from trial 10 (before the response switch) to inform their estimate in trial 11 (after the response switch; Fig. 6*B*). In contrast, at all other time points they used previous guesses to inform their current decision. As participants did not use their previous experience in trial 11, we expected that they instead highly relied on the hint presented in trial 11. Regression analysis confirmed this hypothesis showing a peak at trial 11, similar to the peak at trial 1 (Fig. 6*C*). Furthermore, inspired by the initial modeling results on participants’ strategies (Fig. 4), we tested the influence of previous guesses further in the past [two trials (Fig. 4*D*) and three trials (Fig. 4*E*)]. Again, we found that there is mostly no influence across the response switch. In summary, these analyses showed that participants’ behavior after a response switch was not guided by previous experience, obtained in the opposite response condition, thus suggesting a lack of full knowledge transfer across conditions.

One possible explanation for these results is that participants had difficulties in understanding that the hidden target location had remained the same across first and second 10 trials (i.e., between trial 10 and trial 11). To rule out this possibility, we performed a second experiment with an independent set of participants (*N* = 20) where we further emphasized that the target location was the same across all 20 trials (for details, see Materials and Methods). Furthermore, we asked participants at the end of each block whether they were aware that all 20 trials performed so far belonged to the same hidden target. We obtained overall similar results in this experiment (Extended Data Fig. 5-3), as learning performance, as well as information transfer and confidence were disturbed after the response switch between trial 10 and trial 11, although participants were explicitly instructed that all 20 trials belonged to the same hidden target and were also aware of this (Extended Data Fig. 5-3*B*). This ruled out the possibility that the inability to transfer knowledge between response modalities is because of a cognitive misunderstanding of the task.

## Discussion

The aim of the current study was to characterize the dynamics of prior learning and its dependence on the type of motor response used to report choices. We found that participants could learn a sensorimotor prior within a few trials, with the learning time course being mostly independent of the response type (pro-saccades or anti-saccades). By using a model-comparison approach, we further demonstrated that participants relied more on their own guesses from previous trials compared with visual hints provided in previous trials, again, independent of the response type. This suggests that prior knowledge is represented in terms of previous motor actions and not incoming, external information provided by the visual hints. To verify this hypothesis, we tested whether participants could generalize their learned prior knowledge from one motor context to the other, a switch from pro-saccades to anti-saccades or vice versa. We found that switching the response type caused a drop in performance, close to resetting to naive levels, indicating that experience from one response type could not be fully transferred to the other. This was the case even despite explicit instructions and participants’ awareness that pro-saccade and anti-saccade trials belonged to the same hidden target location. Our results suggest that humans learn sensorimotor priors through monitoring their previous motor decisions rather than external sensory hints. The dependence of learning on past motor decisions discourages generalization of the learned knowledge to conditions where a different visuo-motor mapping is needed.

Our findings suggest that prior knowledge is represented in a motor specific manner during early learning, which is in line with previous studies reporting motor specific priors in different paradigms (Roach et al., 2017; Chambers et al., 2019). We could furthermore identify one potential reason for why generalization is not possible in such contexts, as we found that participants do not memorize the external information from previous trials (visual hints in our case), but instead they memorize their own actions in each trial (Fig. 4*E*,*F*). As our task required an estimation in every trial, indicated by either a pro-saccade or an anti-saccade, the memory of each trial’s decision was probably represented as the motor action taken to indicate the guess. This could also explain why tasks which do not require an explicit response via a motor action are more generalizable (Roach et al., 2017). In these cases, the memory from previous trials is potentially formed in a more abstract way, as participants only have to “think” about their decision, but not perform any specific action. The finding that prior knowledge is built on internal decisions, compared with external cues, could therefore unify some previous controversial findings about prior generalization.

Despite the suggested motor-specific formation of prior knowledge, the algorithm to combine previous experiences to inform the current decision seemed to be similar for both tested response contexts (Fig. 4*E–H*). This suggests that there is a general procedure for how humans combine previous experiences. However, whether prior knowledge can generalize or not depends on the specific manner through which previous experience or decisions are stored in memory (e.g., in terms of motor actions or more abstract decisions). In other words, although at an algorithmic level learning is independent of the response modality, the learned information is stored with a format that is specific for each modality. This explanation is in line with the previously proposed dissociation between learning a policy versus knowledge (Chambers et al., 2019), and further narrows the space of testable predictions regarding the neural implementation of these different types of learning, as the well-described neuronal machinery of pro-saccades and anti-saccades (Munoz and Everling, 2004; Ford et al., 2005; Juan et al., 2008) could allow a characterization of how the two types of learning occur in the brain, for instance through using neuroimaging techniques.

Our study is different from previous studies as it does not test prior learning in a condition where there is also sensory uncertainty (Berniker et al., 2010; Dekleva et al., 2016). In these task designs, participants are asked to perform a task trial-by-trial and are not explicitly told to combine information across trials. Prior learning in these cases is therefore implicit and potentially unconscious. Furthermore, learning is mostly observed by analyzing how participants combine the noisy sensory information in a given trial with the formed prior. It is therefore not directly possible to resolve which of the two is learned, as observed changes in this combination could potentially come from a changed likelihood distribution, from a changed prior distribution, or from changes in both distributions. Because of these limitations, we designed our task such that the sensory information in each trial is given by a clearly visible hint. We then explicitly asked the participants to combine the information across trials to find the hidden target location. Compared with previous prior learning studies (Berniker et al., 2010), we could therefore directly examine the ability to learn statistical regularities over trials. This more general statistical learning context has also been studied previously, for example showing that learning can happen rapidly within a dozen trials if feedback is provided (Chukoskie et al., 2013). However, to our knowledge, pure statistical learning, without trial-by-trial feedback, together with generalization has not been studied in this context.

The two different task contexts we investigated in this work are distinct to previous studies, as we did not test generalization from one effector to another, such as performing a task with the right hand and switching to the left (Hewitson et al., 2018; Kumar et al., 2020), or switching from a motor to a perceptual task (Chambers et al., 2019). Instead, our two contexts represent two distinct cue-action mappings, though performed with the same motor system (oculomotor). By cue-action mapping we mean that participants had to indicate their guess (the internal cue) with two different response types, pro-saccades and anti-saccades (the actions), dependent on the task context. Potentially, generalization could be easier between different cue-action mappings compared with generalization between different effectors or tasks. In fact, previous studies have shown that probabilistic manipulations employed in either pro-saccades or anti-saccades affect both response types, suggesting that some form of statistical information is transferred across the two response modalities (Liu et al., 2010; Chiau et al., 2011; Pierce et al., 2015), albeit these studies have employed a task where statistical uncertainty is relatively low and does not have to be actively learned. Given the specific design of our task, we cannot differentiate whether participants form a motor independent spatial prior, which is aligned to the given cue-action mapping, or whether they form their prior directly at the motor level. In both cases generalization would fail, matching our experimental observation. Potentially, participants learn a spatial representation in the pro-saccade context, where the correct estimate lies close to their performed motor action endpoint. Then, in the anti-saccade context, they do not follow this estimate and solely invert their motor plan, but instead they form a “pro” representation of the hidden target location in this new context, where again the performed motor action endpoint is close to the acquired spatial estimate. In this interpretation, anti-saccades are not real anti-saccades, but pro-saccades relative to the participants’ estimate and only visual information is inverted. What speaks for this interpretation is the fact that participants seemed to be closer to the optimal learning performance in the anti-saccade condition (Fig. 3*F*,*G*), although this was only the case when the visual hints were narrowly distributed. Interestingly, the only instances where some degree of generalization across response contexts was observed (i.e., when performance after response switch was better than naive levels) occurred when participants performed the hard task condition. Since higher uncertainty in our task discourages a close mapping between motor actions and visual cues (participants learn that the actual cue location is not necessarily near the visual hint), it may instead enable a more context-independent integration of information and formation of abstract knowledge, thereby promoting generalization. However, this abstraction seemed to be incomplete as performance was still affected by a change in response context.

Previous studies examining the effects of statistical regularities in the oculomotor system have primarily focused on how learned statistical information influences saccadic parameters, whereas the involvement of saccades during the learning process itself has been largely ignored. One line of research has investigated how the probabilistic information related to the frequency of appearance of the target in one out of a fixed set of possible locations affects saccadic parameters, both for pro-saccades (Carpenter and Williams, 1995) and anti-saccades (Koval et al., 2004; Liu et al., 2010). These studies have reported faster and more accurate gaze shifts toward high- compared with low-probability locations, and neurophysiological recordings have shown that these behavioral effects are because of an enhanced buildup of neuronal activity preceding saccades toward high-probability locations, observed in the superior colliculus (SC) and frontal eye fields (FEFs; Basso and Wurtz, 1997; Dorris and Munoz, 1998; Everling and Munoz, 2000; for evidence from human neuroimaging studies, see also Liu et al., 2011). Another line of research has investigated how the relative probabilities of pro-saccade versus anti-saccade response types affect saccadic parameters, showing for instance that increasing the relative probability of anti-saccades versus pro-saccades may eliminate the typical anti-saccades’ cost (Chiau et al., 2011; Pierce et al., 2015). The latter probabilistic effects were shown to be associated with modulations in cognitive control regions of the brain (Pierce and McDowell, 2016). Both location and response type probabilistic effects have been reported in settings where statistical uncertainty is relatively low and does not have to be actively learned. One novel aspect of the current study is to test how spatial priors can be learned through eye movements under more complex settings where the possible target locations are not fixed, and probabilities could only be inferred through tracking information over time, which to the best of our knowledge has not been investigated before. Furthermore, pro-saccades and anti-saccades in our study were not tested in terms of how they are affected by the end result of statistical learning, but were examined with respect to how they are involved in active accrual of statistical information from the environment. Despite these differences, our findings are overall in line with the general conceptual framework put forward by previous studies, namely that statistical information is faithfully encoded in the oculomotor system. Future studies will be needed to elucidate where in the oculomotor system neural responses exhibit a similar learning dynamic compared with the effects we observed here. We can, however, speculate that our effects are likely to be reflected in the same areas where location probability effects have been previously reported, namely SC and FEF, although the pattern of learning-related neural modulation might differ from a simple change in neuronal preparatory responses. Our findings also inspire future neuroimaging studies that are shown to be well-suited for differentiation of brain networks underlying pro-saccades versus anti-saccades (Munoz and Everling, 2004; Ford et al., 2005; Juan et al., 2008) and can be extended to contrast brain activations when statistical learning occurs through pro-saccades versus anti-saccades. This will allow to elucidate whether a learned statistical prior in one response modality is locally stored in networks specialized for that modality, as our findings suggest, or is represented by a distributed code encompassing brain areas that underlie both saccadic response types.

Our setup allowed us to simultaneously evaluate participants’ performance, as well as their confidence in their given estimation. Interestingly, confidence also decreased after the response type switch, suggesting that participants were aware that they cannot generalize between both contexts. On the other hand, a control experiment, where we explicitly asked participants to indicate after each block whether they were aware that both response type contexts belonged to the same hidden target estimation, showed that that they knew that information could be combined across both contexts (Extended Data Fig. 5-3*B*). Together, these results suggest that participants’ inability to transfer knowledge from one response context to the other was not because of conscious misunderstanding, but more likely because of the specific mechanisms of how the prior is formed unconsciously.

Although our results, especially the inability to generalize across different motor contexts, suggest that statistical learning is implemented in a low-level, motor-dependent way (Figs. 5, 6), there might be potentially alternative explanations for why we could not see generalization in our experiment. In the following we will discuss these alternative interpretations. One potential explanation for the lack of generalization could be an interference between the internal memory of the target location, which is disturbed by the information slide, presented between trial 10 and trial 11, indicating response switch. Another possibility is that participants would need to train on our task for more than one session, such that they can learn to adapt their way of forming the prior knowledge to something which allows for generalization across response contexts. Furthermore, in our experiments learning blocks were short and participants learned the prior distribution of the target within only 10 trials. To the best of our knowledge, the speed with which statistical regularities can be learned and its relation to the generalization across contexts has not been extensively investigated in the past. However, a few elegant studies have shown that statistical learning can occur within a similar timescale as investigated here. For instance, Turk-Browne et al. (2009) showed that learning-related changes in brain activity start to emerge after the first few repetitions of visual statistical regularities. It has also been shown that the mean compared with the variance of prior distributions can be learned rapidly, within only a few trials (Berniker et al., 2010). Based on these previous results and our current findings, we predict that some properties of the statistical priors can be learned very rapidly in most environments. However, it is possible that the short timescale of only 10 trials is not enough to form an abstract representation of the estimated target location that can be generalized across different contexts. Potentially, initial learning is motor-dependent, but over time this is transformed to a motor-independent knowledge, which can then be transferred to other motor contexts. We also note that in our experiment, switches in response modality were fully predictable as they always occurred after the first 10 trials of each block. This design was employed to reduce participants’ uncertainty regarding the required response, as predictable task switches are in general associated with lower switch costs (Monsell, 2003), and in the specific context of pro-saccades and anti-saccades it has been shown that switch costs between response types is minimal when response types are predictable, i.e., in blocked compared with interleaved designs (Zeligman and Zivotofsky, 2017). It is therefore possible that environments in which changes in response context occur unpredictably or have to be inferred from past information (Sherman and Turk-Browne, 2020) will warrant a more protracted learning of statistical information and higher switch costs. Finally, one major reason for a motor-context dependent learning could be that we did not provide external feedback to guide the learning process, as done in some of the previous studies (Chukoskie et al., 2013). Instead, participants had to rely solely on their internal feedback, potentially coming from the motor system. Future studies will be needed to test these possible explanations.

Spatially directed movements such as saccadic eye movements and reaching are an integral part of our daily activities and acquired skills (e.g., imagine a cellist or a tennis player). Both types of movements are profoundly influenced by statistical priors (Körding and Wolpert, 2004; Brodersen et al., 2008). Furthermore, saccadic eye movements provide detailed sensory information about a scene and are tightly linked to the allocation of attention, hence being instrumental for active vision (Munoz, 2002; Wurtz, 2015). In comparison to the saccadic eye movement, reaching movements have enjoyed a more rigorous characterization of the learning dynamics (Berniker et al., 2010; Fernandes et al., 2014; Chambers et al., 2019; Yin et al., 2019). Inspired by these studies, we characterized the dynamics of statistical learning through eye movements, that are a more accessible motor plan to be tested in the laboratory, and the learned information acquired through their execution can directly impact the very way that the brain samples sensory information (Parr and Friston, 2017).

## Synthesis

Reviewing Editor: Ifat Levy, Yale University School of Medicine Decisions are customarily a result of the Reviewing Editor and the peer reviewers coming together and discussing their recommendations until a consensus is reached. When revisions are invited, a fact-based synthesis statement explaining their decision and outlining what is needed to prepare a revision will be listed below. The following reviewer(s) agreed to reveal their identity: Anouk de Brouwer. Note: If this manuscript was transferred from JNeurosci and a decision was made to accept the manuscript without peer review, a brief statement to this effect will instead be what is listed below.

In this paper, the authors use a novel experimental paradigm to probe how statistical rules are used to inform actions. Specifically, human subjects viewed visual hints indicating the possible location of a “hidden treasure” on a monitor. After viewing the hint, they performed either a pro-saccade or anti-saccade to indicate where they thought the hidden target was. The advantage of this paradigm is that it should be relatively straightforward to detect if the subjects are integrating the hints over multiple trials, and by analyzing learning across switches between pro- and anti-saccades, one should be able to discern if learning generalizes across different motor behaviors. The authors find that a model that incorporates participants previous actions, rather than previous visual information, best explains participants’ behavior. They also find little to no transfer of the learned prior when switching from a pro-saccade to an anti-saccade task, or vice versa. They conclude that humans form sensorimotor priors by using information about motor actions rather than external sensory cues.

The manuscript is well written, the experimental paradigm is intriguing, and the data provide novel insight into learning of statistical priors, and are potentially of high interest to the community. However, the conclusions and the analysis are at times underdeveloped or confusing, as detailed below. These issues limit the impact that the study might have on the field at large.

- Did the authors consider a model that estimates the number of previous trials that participants used to perform their action, rather than comparing 1 to 3 vs all 10 trials in the first half of the block? In theory, participants could use any number in between, and/or they could weigh recent trials more heavily than trials that occurred longer ago.

- The 4 potential strategies outlined in the methods are different from the models described in the results, this is somewhat confusing. Figures 3 and 4 also seem inconsistent in the choice of models. Were the ‘cumulative’ models indeed fitted using the first half (10 trials) of each block, before the switch between saccade direction occurred?

- The authors choose to use only the data from trial 4 onward (l. 292-294) to test the influence of up to three trials in the past on the current trial. This seems like an important choice, as this leaves only 7 trials per block, and learning early in the block (where the learning curve appears to be the steepest) is ignored.

- In the results section, could the authors comment on the individual model fits? Was it evident that all participants used the same strategy?

- The title of figure 3 is that learning curves are stereotypic across response modalities, but it’s unclear what the authors mean by stereotypic. Do you mean that the curves are more similar than you would expect given modality differences in the calibration task? It is also confusing because the authors sometimes use t-tests and sometimes linear mixed models. The use of ‘this difference’ in line 391 is confusing, as the authors were talking about standard deviations, while the modality difference index is calculated using the absolute angular error. The authors then jump back to the effect of modality on learning in the paragraph starting on line 414, after discussing the effect of task difficulty. Why do the authors use median absolute errors here but average absolute errors in the previous section? To make their point, the authors should plot the modality difference as a function of trial number per Figure 3D. On the same plot, show the modality difference for each trial number for the calibration task (with, e.g., a 95% confidence interval). This will directly show what the authors are presumably arguing.

- A heading in the main text (l 443) is “Learning is suboptimal", yet the learning in Figure 3G appears to be very close to optimal. This is mentioned in the discussion, yet there is no clear argument for why this should be the case. What is the interpretation? Perhaps the subjects have a performance ceiling around 10 degrees for the hidden task? The authors need to clarify this in the main text since it contradicts the title of a section in the main text.

- In Figure 4, did the authors combine their easy and hard datasets? If so, the authors need to state why this is justified given the differences seen in easy/hard in Figure 3. Combining easy and hard could muddle the effects seen in Figure 3 (e.g. anti-easy responses look close to the cumulative average hint). If there is sufficient data, the authors need to break up the model comparison into pro/anti easy/hard.

(a) Moreover, the authors should show how model fits compare to the actual responses on held-out blocks. If you fit the best model according to BIC on all blocks except for one, how well do the results match? This would be useful to know given what’s shown in Figure 3.

(b) Figure 4G, was the t-test performed separately on previous guesses and hints? Previous guesses look more different across modalities than the hints.

- Figure 5 is confusing since the authors show an example block in Figure 5A and Figure 1A where the subject generalized across the two modalities (it is assumed that the same data are shown across these two figures, as they look quite similar, probably worth mentioning in the legend). Additionally, some generalization is evident in Figure 5F in the pro-->anti hard transition, this is also clear when the experiment was replicated. Again, the data are at odds with the general conclusion stated by the authors.

(a) The authors mention that the motor error is subtracted from these curves. Was the motor error calculated per trial? More importantly, was the motor error after a switch used for this subtraction? Is it possible that the motor error substantially increases after a modality switch, which would mask generalization after the response switch? This is important to check and could change the interpretation of the data.

(b) What’s going on in Figure 5F rightmost panel? There appears to be a large offset between anti and pro, is this a result of the subtraction? Again, this suggests that on the hidden task the subjects are hitting a performance ceiling, and the offset is due to subtracting off a larger motor error for anti-saccades.

- Figure 6: it is unclear if the analysis demonstrates what is claimed in the title of the figure. While the regression weights for previous guesses decreases substantially after a switch, it seems that the weight would need to be zero for there to be “no” knowledge transfer. If the regression weight dips to, e.g., .25, does that mean there is “no knowledge transfer”? It seems that the answer is no. Here, there appears to be a tendency for previous guesses to be used in the hard condition (especially evident in D,E where the regression weights appear to be significantly above 0). The authors should be clear on what they mean by the title of this figure, or the language should be tempered to reflect what is actually shown: subjects weight visual information more than previous guesses after a switch in a condition-dependent manner (pro/anti easy/hard).

- Given the short learning blocks and participants’ knowledge about when the hidden target changed, could the authors comment on the generalizability of the results to other environments/tasks?

Minor comments

- Consider changing the title - ‘sensorimotor learning’ is a broad term that is not typically used for statistical learning that is investigated in this study.

- Methods: The choice of which equations to present seemed somewhat arbitrary. For example, there are no equations for the models themselves, but there is an equation for the Bayesian weights.

- L. 155-164: Regarding the differences between experiment 1 and 2, did the hidden target also remain the same across a block of 20 trials (and then changed) in experiment 1? This is not explicitly stated in this section. It is a bit difficult to understand the differences between the two experiments.

- L. 178-181: How did participants indicate their confidence level? E.g., did they provide a verbal response or use the keyboard?

- L. 228: By “circular threshold", do the authors mean radius?

- L. 234-245: It would be useful to provide a brief description of the analysis of the gaze data. How was the start of the 500 ms determined? Did the analysis control for whether the eye was fixating or moving during the 500 ms? Were there any requirements on the distance of the fixation position other than having crossed the threshold of 5.375 deg?

- It would also be useful to include how many trials were excluded here or in the results.

- L. 280-283: This would be easier to read if the language was consistent, e.g., by using ‘looking’ as the first word to describe each strategy, or by using ’using’ for the strategies that now start with ‘looking’. Also consider changing ‘visual hints’ in strategy 1 to ‘visual hint’ to clarify that a single hint is used.

- Results: please indicate the actual p-value instead of n.s. when the result is not significant.

- L. 378-380: it seems that a bias is not expected here, because the errors are averaged across targets and participants. It is not clear that it’s useful to test for one.

- Some of the references to Figure 3 seem to be incorrect.

- Table 1. Could the authors also include the mean absolute angular error in the table?

- L. 403-404: The interaction effects don’t indicate that there is only a difference in the hard condition, the t-tests do. Please correct this sentence.

- L. 458: “see Eq 2.” - equation 2 concerns the calculation of the BIC, this reference seems incorrect.

- The relation between the analysis in Figure 3 F-J and Figure 4 is confusing. It seems that the lower bound model is identical to the cum. avg. hint model. However, the limited memory bound model takes into account two recent hints, while the hint model takes into account a single hint and the prev. hint model takes into account three visual hints. Why the inconsistency? Furthermore, Fig 3 F-J and Fig 4A seem to show similar information. Would it make sense to include the cum. avg. guess model in Fig 3 F-J? The authors should also consider including 3F-J with Figure 4.

- L 534-535: Was there a difference in performance between trials 10 and 11 in the calibration blocks? Is it possible that there is a (small) general effect of switching on motor performance?

- Discussion: Could the authors comment on transfer of learning/prior information between pro-saccades and anti-saccades in other tasks? To what extent is this a specific case? Where in the sensorimotor system would this information be stored? Do the authors expect transfer between saccades and reaches, for example?

- There are typos throughout the manuscript, for example:

a. Line 462 “suboptimal” should be “suboptimally"

b. Line 498 “fitted” should be “fit"

c. Line 678 “distribution” should be “distributed"

d. Line 712 “more” should be “higher"

e. Line 723 “the sensory information” should be “sensory information” or “the sensory environment"

- Figure 1B: was not possible to read the orange text without zooming in “Pro-saccades"

- Figure 3D: put the legend here rather than 3E

- Figure 3D: assuming motor error was combined across trials, did the authors estimate if there were any differences across trials?

- Figure 3F-J: hard to discern lower bound vs limited memory bound since they use the same confidence interval color

- Figure 4E: put legend in this panel

- Figure 5F: missing legend (pro/anti)

- Figure 5: the color of the stars is extremely hard to see due to the thick black border. Remove the border and ensure that the different colors are visible at a reasonable zoom level.

- Most of the hypothesis tests used in the manuscript assume normality, the authors should comment on whether this is justified with the type of data they are working with. Also, the authors use a lot of t-tests. For some of the analyses, a linear model might be more appropriate.

- The authors refer to supplementary videos in the manuscript, which was not uploaded.

- It is unclear if Figures S1 or S2 add much. The summary statistics for Figure S2 could be reported without the scatter plot. According to the manuscript, Figure S3 shows similar results to figures shown in the main text (though it also shows some effects that contradict some of the author’s claims, e.g. Figure S3A Hard pro-->anti and easy pro-->anti show apparent savings in learning across the modality transition. Figure S4 could easily be excluded and described in the methods. Figure S5 is potentially interesting, but the authors should be clear on what data is being modeled here. Does this combine easy/hard and pro/anti-saccade data? Why combine here?

## Author Response

Synthesis of Reviews:

Computational Neuroscience Model Code Accessibility Comments for Author (Required):

The authors briefly mention a MATLAB package for analyzing the gaze data, but for the remaining analysis they only mention that R and Python were used. More details regarding the statistical and modelling packages used would be helpful.

Significance Statement Comments for Author (Required):

Appropriate

Comments on the Visual Abstract for Author (Required):

N/A

Synthesis Statement for Author (Required):

In this paper, the authors use a novel experimental paradigm to probe how statistical rules are used to inform actions. Specifically, human subjects viewed visual hints indicating the possible location of a “hidden treasure” on a monitor. After viewing the hint, they performed either a pro-saccade or anti-saccade to indicate where they thought the hidden target was. The advantage of this paradigm is that it should be relatively straightforward to detect if the subjects are integrating the hints over multiple trials, and by analyzing learning across switches between pro- and anti-saccades, one should be able to discern if learning generalizes across different motor behaviours. The authors find that a model that incorporates participants previous actions, rather than previous visual information, best explains participants’ behaviour. They also find little to no transfer of the learned prior when switching from a pro-saccade to an anti-saccade task, or vice versa. They conclude that humans form sensorimotor priors by using information about motor actions rather than external sensory cues.

The manuscript is well written, the experimental paradigm is intriguing, and the data provide novel insight into learning of statistical priors, and are potentially of high interest to the community. However, the conclusions and the analysis are at times underdeveloped or confusing, as detailed below. These issues limit the impact that the study might have on the field at large.

We thank the editor for giving us the opportunity to revise our manuscript and for the overall positive evaluation of our work. We are also very thankful to the reviewers for their insightful suggestions. The editor’s guidance for the revision and the reviewers’ comments have been most valuable and helpful. We have addressed the issues highlighted by the editor and the reviewers in detail. To answer reviewers’ suggestions and concerns we adapted the manuscript at multiple locations, by adding additional analyses, clarifying our methodology and refining our conclusions, as can be seen in the point-by-point reply below. We believe that these changes have made our paper much stronger and we hope that our manuscript will be suitable for publication in the current form.

The editors’ and reviewers’ comments are in blue, our responses are in black and text excerpts from the main manuscript are in red font colour. Changes to the manuscript based on reviewers’ suggestions are also marked in red in the manuscript document.

Point-by-point reply

1) Did the authors consider a model that estimates the number of previous trials that participants used to perform their action, rather than comparing 1 to 3 vs all 10 trials in the first half of the block? In theory, participants could use any number in between, and/or they could weigh recent trials more heavily than trials that occurred longer ago.

We thank the reviewer for pointing this out. To estimate the number of trials in the past, we performed a stepwise linear regression including all information (previous guesses + current and previous visual hints) available to participants to inform their action, measured in terms of angular error in trial 10 (new extended figure 4-2, see below). This analysis showed that 1) including more than six predictors (combinations of previous guesses and visual hints) did not improve the model accuracy for most subjects (Fig. 4-2 C), and that 2) the best model in most participants contained the current visual hint as well as the three previous guesses as predictors (Fig. 4-2 D). Based on these results, the model comparison throughout was focused on models including three timesteps in the past.

We have now added this information to page 19, lines 479-494:

Adapted text in the main manuscript:

In a second step, we tested several multi-predictor models to get a more detailed view on participants’ learning strategy. To determine the number of trials in the past potentially having an influence on the current decision, we conducted a stepwise linear regression analysis, where all previous guesses and all presented hints thus far were included to estimate participant’s angular error in trial 10 (Fig. 4-2). This showed that for most participants, models with a low number of predictors performed as well as or better than models including all past trials (Fig. 4-2 A-C). Furthermore, we found that the predictors most often included in the best model were the current visual hint and the three previous guesses (Fig. 4-2 D). Based on these results, we chose to include information from up to three timesteps in the past and specifically compared five different options of how previous guesses and presented visual hints can be combined (Table 1). The model comparison revealed that multi-predictor models which relied on combinations of external and internal information, i.e., visual hints and previous guesses, performed much better than models that were based on only one of the two sources of information (i.e., either the previous three guesses or the current and previous three visual hints) (Fig.4 B). This was consistent across subjects (Fig. 4-4). Again, splitting the data into pro- and anti-saccade blocks did not affect the main trend (Fig.4 C-D), nor did splitting the data into hard and easy task difficulty (Fig. 4-5).

Figure 4-2: Stepwise regression to select relevant predictors for model comparison.

This figure is supplementary to Fig.4 in the main text. Here, we used stepwise linear regression (train function with method “leapForward” from the Caret package in R) to estimate the relevant number of past trials that should be included in our main model comparison analyses (Fig.4 & Table 1). We tried to predict the angular error in trial 10 from either all previous guesses (A), all observed visual hints (B), or a combination of both (C-D). Shown is the cross-validated fit accuracy (10-fold), measured as the root-mean-squared error (RMSE). Thereby, we found for each individual subject the number of trials (=predictors) that were included in the best fitting model (red dot), indicated by the lowest RMSE. To identify not only the number, but also the type of information that best predicted performance in trial 10, we analyzed which timesteps of previous guesses and current and previous hints were included in the best combined model (C) and created a histogram indicating for how many subjects the specific predictor (either guess: G or Hint: H) was included in the best model (D).

Using this approach, we found that for most subjects a model with 6 or less predictors, including a combination of previous guesses and visual hints, is best in predicting the angular error of the current trial. Thereby, we focused our main analysis (Fig.4 & Table 1) on three timesteps in the past (up to t-3). This allowed us to predict not only the behavior in trial 10, as done here, but also the behavior from trial 4-10. A general trend in the main analysis, which is also apparent here, is that previous guesses are better in predicting current behavior, compared to current or previous visual hints (lower errors for models including guesses (A) compared to hints (B)).

2) The 4 potential strategies outlined in the methods are different from the models described in the results, this is somewhat confusing. Figures 3 and 4 also seem inconsistent in the choice of models. Were the ’cumulative’ models indeed fitted using the first half (10 trials) of each block, before the switch between saccade direction occurred?

Thanks for this comment. Indeed, the modelling method section was poorly described. We tried to improve this by clearly explaining (with equations) all models which are listed in Fig.4, as can be seen in Table 1 on page 11, and thoroughly revising the text in the modelling method section (pages 10-11, lines 294-315). All modelling results are now depicted in Figure 4, whereas in Figure 3, we only show the theoretical lower bound on performance (which is not a model fitted to subjects’ data, but instead comes from theoretical considerations) (Figure 3F-J). Regarding the question about the ’cumulative’ models, we used indeed the first 10 trials and calculated for each trial a cumulative average estimate (e.g., for trial 3 we calculated the cumulative average of presented hints on trial 1-3). These values were then included as predictors in a linear regression (as described in the modelling methods section and Table 1). To be able to compare fit accuracy between all models presented in Table 1, we had to only use data from trial 4 until 10, such that all models could be trained on the same data. This is now clarified in the text on page 11, lines 307-308.

Adapted text in the main manuscript:

Modeling participants’ behavior

We used linear regression models (lm package in R) to analyze the single subject behavior. To this end, the angular error of participants’ estimate in a given trial (i.e. their guess in trial t, Guesst) was fitted by models having a diverse set of independent variables as shown in Table 1.

The five single predictor models we tested were 1) using the visual hint in each trial, 2) using the visual hint in the preceding trial (t-1), 3) using the cumulative average of all visual hints so far, 4) using the guess from the previous trial, 5) using the cumulative average of all previous guesses (Fig.4 A & Table 1). The dependent variable was the angular error of a participant’s estimate in a certain trial (Guesst). The independent variable was the angular error of the estimate, given by one of the above-mentioned strategies. In a second stage, we tested linear regression models with multiple independent variables. For this, we included previous guesses from up to three time steps in the past, as well as the visual hints from the current and up to three time steps in the past. Our choice of including three trials in the past was inspired by a naïve model search approach (Fig. 4-2), which indicated that including up to 3 trials in the past provided the best fit to the participants’ data. Thus, in our multi-predictor models only the data from trial 4 to trial 10 was included, allowing all models to be tested on the same data. Each of the described regression models was fitted to the single subject data using R’s lm package. To compare the models, we calculated BIC, delta BIC, and Bayesian weights (Burnham and Anderson, 2004), to assess the likelihood of each model being the best fit to the data (cf. Evaluating model performance). Since these values are normalized, they can be used to determine the model that on average best fits the participants’ data.

Table 1. Overview of the models used to assess sensorimotor statistical learning.

3) The authors choose to use only the data from trial 4 onward (l. 292-294) to test the influence of up to three trials in the past on the current trial. This seems like an important choice, as this leaves only 7 trials per block, and learning early in the block (where the learning curve appears to be the steepest) is ignored.

We understand this concern. However, we note that the central aim of the current study was to investigate how past information is used to build a prior for current decisions. As such, we had no choice but to start our modeling at least after trial 1. The exact number of trials in the past to be included in our models (n=3) was determined based on a stepwise linear regression analysis (Figure 4-2). This way, we were able to have a consistent dataset across models (including those looking at several time steps in the past). To nevertheless address the reviewer’s point, we compared the models from our model selection in Fig.4 which only need one timestep in the past, which allowed us to use all trials except the first one. This showed similar results to what we found in Fig.4, although the model using only hints performed a bit better, which is expected in earlier trials (new extended figure 4-3).

Figure 4-3: Model comparison results of single predictor models tested on trial 2-10.

This figure is supplementary to Fig.4 B in the main text. Our main modeling results were based on models that included up to three timesteps in the past (n=3), where n was determined based on a model search approach (Fig. 4-2). To this end, we included trials 4-10, allowing us to test the models on a consistent dataset. To test whether our results hold when we include earlier trials in a block, we repeated our analysis focusing only on single predictor models (Table 1 models 1-5), which allowed us to use the data from trial 2-10. Similar to our main results, the best single predictor model was the one based on the cumulative average of past guesses.

4) In the results section, could the authors comment on the individual model fits? Was it evident that all participants used the same strategy?

To address this point, we added the single subject model comparison results as a new extended figure (new extended figure 4-4). Subjects indeed consistently used similar strategies, meaning that there was no single subject where the best model consisted of only using visual hints and not previous guesses. Most of the subjects relied on a combination of visual hints and previous guesses.

Figure 4-4: Model comparison results for single subjects.

This figure is supplementary to Fig.4 B in the main text. Here, we show the model comparison results for each single subject individually.

5) The title of figure 3 is that learning curves are stereotypic across response modalities, but it’s unclear what the authors mean by stereotypic. Do you mean that the curves are more similar than you would expect given modality differences in the calibration task? It is also confusing because the authors sometimes use t-tests and sometimes linear mixed models. The use of ’this difference’ in line 391 is confusing, as the authors were talking about standard deviations, while the modality difference index is calculated using the absolute angular error. The authors then jump back to the effect of modality on learning in the paragraph starting on line 414, after discussing the effect of task difficulty. Why do the authors use median absolute errors here but average absolute errors in the previous section? To make their point, the authors should plot the modality difference as a function of trial number per Figure 3D. On the same plot, show the modality difference for each trial number for the calibration task (with, e.g., a 95% confidence interval). This will directly show what the authors are presumably arguing.

We appreciate this comment and agree that this section was confusing as we jumped back and forth between standard deviation and absolute angular error. To improve this, we extensively reformulated the whole section, focusing solely on the analysis of the absolute angular error, as this is the measure we were eventually interested in and what we used in the rest of the manuscript (lines 385-417). To remedy the confusion with the word ’Stereotypic’, the title of figure 3 and the corresponding section were changed to ’Similarity of learning curves across response modalities during the statistical learning’. In addition, we also exchanged the modality difference figure as suggested by the reviewer (see Figure 3c also inserted below).

Adapted text in the main manuscript:

Similarity of learning curves across response modalities

To work out whether statistical learning is similar across response modalities, we contrasted the learning performance in pro- versus anti-saccade trials in the hidden target task, as well as in the calibration task. First, we calculated the mean and the standard deviation of the respective angular error distributions, pooled across participants (Fig.3 A&B and Table 2), as well as the performance measure used here and in the following - the absolute angular error (Table 2).

Table 2: Angular error distribution for the calibration and the hidden target task. Mean and standard deviation for distributions shown in Fig. 3A&B. The rightmost column indicates the average absolute angular error in each task condition, estimated from the subjects’ guess, or, for the last row, from the distribution of visual hints.

For both modalities and both task difficulties, the absolute angular error in the hidden target task was higher than in the calibration task (pro hard: t=13.8, p<0.0001; pro easy: t=8.8, p<0.0001; anti hard: t=14.1, p<0.0001; anti easy: t=2.9, p=0.0084; paired t-test each with N=20). This makes sense as in the former task the target location was uncertain whereas in the latter it was not. Furthermore, the performance in pro- and anti-saccade blocks was only significantly different during the calibration task, but not during the hidden target task (calibration task: t=5.6, p<0.0001; hidden target task easy: t=1.7, p=0.1106; hidden target task hard: t=1.8, p=0.0903; paired t-test each with N=20). To quantify this difference further, we calculated a modality difference index (described in Materials and Methods section), which was consistently higher in the calibration task compared to the hidden target task (Fig.3 C). These results provide first evidence that statistical learning is similar for pro- and anti-saccade response.

So far, we have shown that the average performance in the first ten trials does not seem to depend on the modality used for indication - pro- or anti-saccades. Nevertheless, the specific time course of statistical learning could still be modality dependent. For this, we next looked at the performance in a trial-dependent manner (Fig.3 D). As expected from the analysis in Fig.2 C, we observed that the performance is slowly increasing with each trial, indicating that the participants were using the information given in each trial to improve their estimate of the hidden target location. This general behavior was also reflected in the time course of subjects’ own confidence (Fig.3 E). In line with the previous, trial-independent analysis, we found that the performance for pro- and anti-saccades was very similar (Fig.3 D cf. red versus blue). This similarity seemed especially interesting when compared to the observed difference in motor error during the calibration task (Fig.3 D dashed line). In summary, inspection of the learning curves of pro- and anti-saccade indication supported our initial results suggesting that statistical learning is independent of the response modality.

Figure 3 C.

6) A heading in the main text (l 443) is “Learning is suboptimal", yet the learning in Figure 3G appears to be very close to optimal. This is mentioned in the discussion, yet there is no clear argument for why this should be the case. What is the interpretation? Perhaps the subjects have a performance ceiling around 10 degrees for the hidden task? The authors need to clarify this in the main text since it contradicts the title of a section in the main text.

We thank the reviewer for pointing out this discrepancy. Indeed, the fact that we found close to optimal behaviour in the anti-easy condition was puzzling. To address this important comment, we added a paragraph discussing it at the end of the corresponding section in Results, on page 18-19, lines 449-461.

One possible explanation for this effect is that the easy task condition could potentially allow for optimal learning, as the visual hints are narrowly dispersed around the mean, i.e. the actual target location. It is however possible that this optimal learning is disturbed in the case of pro-saccade responses, as visual hints are very close to the participants’ estimates based on statistical learning and could partially ’distract’ participants to direct their gaze towards the location of the hint rather than towards the estimated mean of the distribution. However, in anti-saccades, this ’distraction effect’ occurs to a much lesser extent, as participants need to plan their responses not towards but opposite to the location of the visual hints. Due to a weaker distraction, participants may be able to more reliably indicate their estimation of the distributions’ mean rather than following the individual samples, and hence perform nearly optimally. This could be a potential factor leading to a performance ceiling as the reviewer suggested.

Adapted text in the main manuscript:

Interestingly, we found minor differences in terms of performance between the four different conditions we tested (pro/anti x hard/easy). Three out of four conditions showed clear suboptimal behavior (Fig.3 F,H,J). However, in the condition where the visual hint distribution was more concentrated (i.e., the easy condition) and anti-saccades were used as the response modality, performance was close to optimal (Fig.3 G). As described before, this finding cannot be ascribed to the fact that our estimate of the motor error was flawed, thereby artificially improving the measured performance, as the estimate for Trial 1 closely matched what we observed in participants’ behavior (Fig. 5-4). We suspect that in this condition, performing an anti-saccade discourages a bias to follow the visually presented lines too much, and hence can be beneficial in producing optimal performance. Nevertheless, the differential learning of pro- and anti-saccade responses with respect to an optimal lower bound on performance was not present in the hard task condition (Fig.3 H-J), indicating that the optimal learning for anti-saccades only occurred when the visual hints where narrowly distributed.

7) In Figure 4, did the authors combine their easy and hard datasets? If so, the authors need to state why this is justified given the differences seen in easy/hard in Figure 3. Combining easy and hard could muddle the effects seen in Figure 3 (e.g. anti-easy responses look close to the cumulative average hint). If there is sufficient data, the authors need to break up the model comparison into pro/anti easy/hard.

(a) Moreover, the authors should show how model fits compare to the actual responses on held-out blocks. If you fit the best model according to BIC on all blocks except for one, how well do the results match? This would be useful to know given what’s shown in Figure 3.

(b) Figure 4G, was the t-test performed separately on previous guesses and hints? Previous guesses look more different across modalities than the hints.

We thank the reviewer for these suggestions. We added a new extended figure showing the model comparison results for each of the four experimental conditions separately (Fig. 4-5, see below) and mentioned it when talking about the strategy analysis (lines 493-494). The main observations in Figure 4, that 1) the best single-predictor model is Cum.avg.guess, and 2) the best multi-predictor models include combinations of previous guesses and observed hints, were consistently observed in all four experimental conditions. This suggests that the general strategy does not majorly vary between the easy and hard task condition. Instead, the pattern of results suggests that participants use the same strategy, but since the distribution of visual hints in hard and easy conditions is different, they arrive at vastly different performance levels as seen in Figure 3.

Regarding (a): the new Fig. 4-5 E now shows the model accuracy in a held-out block for an example session.

Regarding (b): Yes, one t-test was used for previous guesses and one for hints. This is now clarified on page 20 (lines 506-507) and the legend of Figure 4G-H.

Figure 4-5: Dependency of model comparison results on experimental condition.

This figure is supplementary to Fig.4 in the main text. A-D) Model comparison results for data split according to pro/anti-saccades and easy/hard task difficulty. Overall model comparison results did not depend on the response type or task difficulty. E) Example model prediction for a held-out data block.

8) Figure 5 is confusing since the authors show an example block in Figure 5A and Figure 1A where the subject generalized across the two modalities (it is assumed that the same data are shown across these two figures, as they look quite similar, probably worth mentioning in the legend). Additionally, some generalization is evident in Figure 5F in the pro-->anti hard transition, this is also clear when the experiment was replicated. Again, the data are at odds with the general conclusion stated by the authors.

(a) The authors mention that the motor error is subtracted from these curves. Was the motor error calculated per trial? More importantly, was the motor error after a switch used for this subtraction? Is it possible that the motor error substantially increases after a modality switch, which would mask generalization after the response switch? This is important to check and could change the interpretation of the data.

(b) What’s going on in Figure 5F rightmost panel? There appears to be a large offset between anti and pro, is this a result of the subtraction? Again, this suggests that on the hidden task the subjects are hitting a performance ceiling, and the offset is due to subtracting off a larger motor error for anti-saccades.

Thanks for pointing this out. To prevent confusion, we exchanged the shown example block in Figure 5A with a more representative example.

Regarding (a): The motor error was estimated based on the calibration block and not per trial. To address the general concern about whether the drop in performance might be primarily due to a ’pure’ motor error, which increased after a modality switch, we added an extending figure showing that the error in the calibration task does not significantly increase after the switch from pro- to anti-saccades (Fig. 5-4 A). Thus, the drop in performance seen in the hidden target task did probably not come from a motor effect alone. To further check the validity of our assumption that the total error can be separated into a statistical and a motor error, we looked at trial 1 and compared the total error with the estimate predicted from combining the motor error, measured in the calibration task, and the statistical error, calculated from the shown hint distribution in trial 1 (Fig. 5-4 B-F, also shown below). Both matched to a high degree and we could not find any statistically significant difference between the two for any of the conditions (pro-easy; anti-easy; pro-hard; anti-hard).

To address the reviewer’s remark that some degrees of generalization is evident in Figure 5F, we revised our analysis and adapted our conclusions (lines 560-588 in Results, lines 653-655 and 728-736 in Discussion). To additionally check the results we obtained in Fig.5, we added a direct comparison of trial 1 and trial 11 within response modality, where a subtraction of the motor error was thus not necessary (Fig.5 F dashed lines; new paragraph lines 573-588). This confirmed our main conclusion that there was a drop in performance from trial 10 to trial 11.

Regarding (b): Thanks for pointing this out. There was a mistake in the subtraction of the motor error previously. We corrected this in the current version of figure 5 (see Figure 5H).

Figure 5-4: Motor error estimation.

This figure is supplementary to Fig.3 and Fig.5 in the main text. Here, we tested whether our assumption that participants’ estimation error in the hidden target task calculated as a combination of two independent sources of uncertainty; i.e., the motor noise (estimated from the calibration task) and statistical uncertainty (estimated from the distribution of visual hints in the hidden target task), was plausible. A) Time course of motor error in the calibration task. B) In the following we examined Trial 1 of the hidden target task and compared subjects’ actual performance to the theoretical prediction of adding motor noise and statistical uncertainty. If our assumption that both noise sources are independent and thus can be added is plausible, we would expect that the actual (x axis) and predicted (y axis) data would match. C-F) Results for all four experimental conditions. Each dot represents one subject (N=20). Paired t-tests were performed to test whether there is a significant difference between the theoretical prediction and the actual data and results are shown in the respective panels.

9) Figure 6: it is unclear if the analysis demonstrates what is claimed in the title of the figure. While the regression weights for previous guesses decreases substantially after a switch, it seems that the weight would need to be zero for there to be “no” knowledge transfer. If the regression weight dips to, e.g., .25, does that mean there is “no knowledge transfer”? It seems that the answer is no. Here, there appears to be a tendency for previous guesses to be used in the hard condition (especially evident in D,E where the regression weights appear to be significantly above 0). The authors should be clear on what they mean by the title of this figure, or the language should be tempered to reflect what is actually shown: subjects weight visual information more than previous guesses after a switch in a condition-dependent manner (pro/anti easy/hard).

The rationale for the title of Figure 6 was that after the switch in response modality, there was mostly no significant influence of the previous trials on the current one. However, Figure 6 did not demonstrate this clearly. We now explicitly demarcate the non-significant regression weights in Figure 6 (shown below). This suggested that the knowledge prior to the response switch is not used in post-switch trials. However, as the reviewer noted, there is some significant influence across modality switch when looking at trials further in the past (Fig. 6D-E). To reflect this, we tempered the title of this figure to ’Almost no’, instead of ’No’ (line 634).

Figure 6: Almost no knowledge transfer between visuo-motor modalities. (A) To test if there is any knowledge transferred from the experience with one response type to the other, we regressed the current guess against previous guesses/current hint. (B) Participants’ estimates at trial 11 (after response switch) are independent of the estimates at trials 10 (before response switch). In contrast, at every other time point, participants use previous experience to inform their current guess. (C) At trial 11, participants highly rely on the information coming from the current hint. (D-E) Similar to the lack of transfer from one trial to the next (B), there is also no transfer from trials further in the past across the response switch (trial 11-12 for t-2; trial 11-13 for t-3). In B, D and E non-significant regression weights with a p-value>0.05 are shown in opaque.

10) Given the short learning blocks and participants’ knowledge about when the hidden target changed, could the authors comment on the generalizability of the results to other environments/tasks?

We thank the reviewers for this important point. We tried to clarify this by adding the following section to the Discussion, on page 31, lines 794-817.

Adapted text in the main manuscript:

Furthermore, in our experiments learning blocks were short and participants learned the prior distribution of the target within only ten trials. To the best of our knowledge, the speed with which statistical regularities can be learned and its relation to the generalization across contexts has not been extensively investigated in the past. However, a few elegant studies have shown that statistical learning can occur within a similar timescale as investigated here. For instance, Turk-Browne et al. (Turk-Browne et al., 2009) showed that learning-related changes in brain activity start to emerge after the first few repetitions of visual statistical regularities. It has also been shown that the mean compared to the variance of prior distributions can be learned rapidly, within only a few trials (Berniker et al., 2010). Based on these previous results and our current findings, we predict that some properties of the statistical priors can be learned very rapidly in most environments. However, it is possible that the short timescale of only ten trials is not enough to form an abstract representation of the estimated target location that can be generalized across different contexts. Potentially, initial learning is motor-dependent, but over time this is transformed to a motor-independent knowledge, which can then be transferred to other motor contexts. We also note that in our experiment, switches in response modality were fully predictable as they always occurred after the first 10 trials of each block. This design was employed to reduce participants’ uncertainty regarding the required response, as predictable task switches are in general associated with lower switch costs (Monsell, 2003), and in the specific context of pro- and anti-saccades it has been shown that switch costs between response types is minimal when response types are predictable; i.e. in blocked compared to interleaved designs (Zeligman and Zivotofsky, 2017). It is therefore possible that environments in which changes in response context occur unpredictably or have to be inferred from past information (Sherman and Turk-Browne, 2020) will warrant a more protracted learning of statistical information and higher switch costs.

Minor comments

- State which R and Python package were used

We added this information throughout.

- Consider changing the title - ’sensorimotor learning’ is a broad term that is not typically used for statistical learning that is investigated in this study.

Many thanks for this suggestion. We have now changed the title to ’Previous motor actions outweigh sensory information in sensorimotor statistical learning’.

- Methods: The choice of which equations to present seemed somewhat arbitrary. For example, there are no equations for the models themselves, but there is an equation for the Bayesian weights.

We addressed this comment by adding a full list of model equations corresponding to the models tested in Fig.4 in Table 1.

- L. 155-164: Regarding the differences between experiment 1 and 2, did the hidden target also remain the same across a block of 20 trials (and then changed) in experiment 1? This is not explicitly stated in this section. It is a bit difficult to understand the differences between the two experiments.

Yes, the hidden target task itself is identical in experiment 1 and 2, thus also in experiment 1 the hidden target remained the same across a block of 20 trials. To make this clearer, we now have two separate sections in Methods, corresponding to each experiment. We put the section for Experiment 2 at the end of the Experimental setup section and reformulated it to clarify the misunderstanding (page 8, lines 230-240).

- L. 178-181: How did participants indicate their confidence level? E.g., did they provide a verbal response or use the keyboard?

The used the keyboard to indicate their confidence. We added a sentence in the methods to clarify this, on page 6, line 173-174.

- L. 228: By “circular threshold", do the authors mean radius?

Yes, we changed the phrasing accordingly, as can be seen in line 222.

- L. 234-245: It would be useful to provide a brief description of the analysis of the gaze data. How was the start of the 500 ms determined? Did the analysis control for whether the eye was fixating or moving during the 500 ms? Were there any requirements on the distance of the fixation position other than having crossed the threshold of 5.375 deg?

We have now revised the ’Successful response’ section on page 8 (lines 220-228) to clarify how the start of the 500 s window was determined. We used a moving window to calculate how much the gaze data deviated, and as soon as this deviation was below 1 dva the response was counted as successful. Thus, there was no fixed start of the 500 ms, but it was instead defined retrospectively by the end of the successful response. There were no further constraints on the fixation, as e.g., whether the eye was moving or not.

- It would also be useful to include how many trials were excluded here or in the results.

We have now included the number of excluded trials in the Data pre-processing section on page 9, lines 257-262.

- L. 280-283: This would be easier to read if the language was consistent, e.g., by using ’looking’ as the first word to describe each strategy, or by using ’using’ for the strategies that now start with ’looking’. Also consider changing ’visual hints’ in strategy 1 to ’visual hint’ to clarify that a single hint is used.

Thanks for this tip. We carefully checked the manuscript for consistency of the terminology and adjusted the text accordingly.

- Results: please indicate the actual p-value instead of n.s. when the result is not significant.

We have now included all the actual p-values.

- L. 378-380: it seems that a bias is not expected here, because the errors are averaged across targets and participants. It is not clear that it’s useful to test for one.

We agree that such a bias is not expected. Therefore, we excluded this analysis from the manuscript.

- Some of the references to Figure 3 seem to be incorrect.

We checked this but could not find any incorrect references. Potentially they were already resolved after reformulating some paragraphs.

- Table 1. Could the authors also include the mean absolute angular error in the table?

We included the mean absolute angular error in the table (Table 2, page 15).

- L. 403-404: The interaction effects don’t indicate that there is only a difference in the hard condition, the t-tests do. Please correct this sentence.

This section (lines 385-417) was generally rewritten to clarify the presented analysis (no longer jumping back and forth between analysing standard deviation or angular error) and now does not include the referred to analysis anymore.

- L. 458: “see Eq 2.” - equation 2 concerns the calculation of the BIC, this reference seems incorrect.

This is now clarified.

- The relation between the analysis in Figure 3 F-J and Figure 4 is confusing. It seems that the lower bound model is identical to the cum. avg. hint model. However, the limited memory bound model takes into account two recent hints, while the hint model takes into account a single hint and the prev. hint model takes into account three visual hints. Why the inconsistency? Furthermore, Fig 3 F-J and Fig 4A seem to show similar information. Would it make sense to include the cum. avg. guess model in Fig 3 F-J? The authors should also consider including 3F-J with Figure 4.

The main difference between the analysis done in Figure 3F-J and Figure 4 is that the relevant measure changes. The idea was to present the performance, measured as absolute angular error, in Figure 3. We thought it would be useful to have a lower bound here to be able to fairly evaluate subjects’ performance. We agree that the way we presented it was confusing. To clarify this, we now excluded the Limited memory curve from Figure 3 F-J. Figure 3 is now only showing the definitive lower bound, and not going into specific modelling options. In contrast, Figure 4 is modelling the signed angular error, so not the absolute angular error as presented in Figure 3, as here we really wanted to understand how subjects made their choices. We hope this helped to circumvent the confusion about the modelling.

- L 534-535: Was there a difference in performance between trials 10 and 11 in the calibration blocks? Is it possible that there is a (small) general effect of switching on motor performance?

This is an important point. To address this, we included a new extended figure (Fig. 5-4) testing whether there is a time dependent motor error. The error in trial 11 is naturally different from the one in trial 10 as it is performed using a different visuomotor modality. However, what we really want to test is whether the error in trial 11 is significantly higher (or lower) than at the end of the period using the same visuomotor modality, which would then indicate that there is indeed an increased motor error due to the switch itself, which is slowly decaying with more trials of the same visuomotor modality. When we compared the error in trial 11 and trial 20 in the calibration task we could not find any significant difference, as now mentioned in lines 537-540.

- Discussion: Could the authors comment on transfer of learning/prior information between pro-saccades and anti-saccades in other tasks? To what extent is this a specific case? Where in the sensorimotor system would this information be stored? Do the authors expect transfer between saccades and reaches, for example?

Thanks for bringing these discussion points to our attention. We changed several parts of the Discussion (lines 712-716 and new paragraph in lines 737-774) to accommodate these points. Specifically:

Re. ’Could the authors comment on transfer of learning/prior information between pro-saccades and anti-saccades in other tasks? To what extent is this a specific case?’

To the best of our knowledge, most previous studies have been focused on how saccade preparation is influenced by learned statistical information, whereas the role of eye movements in active learning of statistical regularities has been largely ignored. This is partly due to the fact that previous studies employed much simpler probability manipulations compared to ours; either varying the probability of the target appearing in a fixed set of locations or the probability of pro- versus anti-saccades response types; hence minimizing the need for active learning of statistical information. We are not aware of any other study that has characterized the dynamics of learning and the nature of learned information in the oculomotor system. In contrast, studies on reaching movement have characterized statistical learning in more detail and with the use of a computational modelling approach (Berniker et al., 2010; Chambers et al., 2019; Dekleva et al., 2016; Fernandes et al., 2014; Yin et al., 2019), which also inspired the current study (see also the new paragraph, lines 737-774 and 827-829). Regarding the transfer of information across saccade types: previous studies have provided evidence that some form of statistical information is transferred across pro- and anti-saccades (Chiau et al., 2011; Liu et al., 2010; Pierce et al., 2015). However, the statistical uncertainty in these setting was low and learning was not explicitly examined (lines 712-716).

Re. Where in the sensorimotor system would this information be stored?

We have extensively discussed this point: we believe that the statistical information contained in our task is most likely stored in the Superior Colliculus and Frontal Eye Fields, two important nodes of the oculomotor system that have been shown to encode probabilistic information, as detailed in the new paragraph, lines 737-774.

Re. Do the authors expect transfer between saccades and reaches, for example?

The similarity of learning dynamics across pro- and anti-saccades suggests that there is a general algorithm for how encountered statistical information is integrated to build a prior, as mentioned in lines 674-682. This procedure is likely to extend to other motor systems such as reaching movements. However, we also observed a motor-specific representation of learned information which hampered a full transfer of information across pro- and anti-saccades. The latter observation hints to the possibility that statistical learning cannot be generalized across saccades and reaching movements. However, this is a speculation that awaits future studies to be explicitly tested.

- There are typos throughout the manuscript, for example:

a. Line 462 “suboptimal” should be “suboptimally"

b. Line 498 “fitted” should be “fit"

c. Line 678 “distribution” should be “distributed"

d. Line 712 “more” should be “higher"

e. Line 723 “the sensory information” should be “sensory information” or “the sensory environment"

Thanks for pointing these out. We corrected them and additionally did another proof-read.

- Figure 1B: was not possible to read the orange text without zooming in “Pro-saccades"

- Figure 3D: put the legend here rather than 3E

- Figure 3D: assuming motor error was combined across trials, did the authors estimate if there were any differences across trials?

- Figure 3F-J: hard to discern lower bound vs limited memory bound since they use the same confidence interval color

- Figure 4E: put legend in this panel

- Figure 5F: missing legend (pro/anti)

- Figure 5: the color of the stars is extremely hard to see due to the thick black border. Remove the border and ensure that the different colors are visible at a reasonable zoom level.

Again, thanks for pointing these out. We adapted the figures accordingly. We added a supplementary figure testing the motor error in the calibration task in a trial-dependent manner (Fig. 5-4). For the main manuscript we calculated a trial-independent motor error, combining the measurements across trials.

- Most of the hypothesis tests used in the manuscript assume normality, the authors should comment on whether this is justified with the type of data they are working with. Also, the authors use a lot of t-tests. For some of the analyses, a linear model might be more appropriate.

Thanks for this remark. We have now explicitly stated whether the assumption of normality is plausible or not. To this end, we performed Shapiro-Wilk tests on the data tested in Fig 2C-E, which did not show any significant deviation from normality (lines 265-269).

We use t-test on the summary data of each participants, whenever a hypothesis regarding whether participants had in general learned (figure 2C-E). Previous t-tests related to the transfer of information across response modalities are removed as the reviewer suggested and replaced by ANOVAs (Figure 5 and lines 532-588).

- The authors refer to supplementary videos in the manuscript, which was not uploaded.

Thanks for bringing this mistake to our attention. We excluded the reference to supplementary videos from the manuscript, as they do not directly extend any of the presented figures and are therefore not to include according to Eneuro guidelines (as far as we understand).

- It is unclear if Figures S1 or S2 add much. The summary statistics for Figure S2 could be reported without the scatter plot. According to the manuscript, Figure S3 shows similar results to figures shown in the main text (though it also shows some effects that contradict some of the author’s claims, e.g. Figure S3A Hard pro-->anti and easy pro-->anti show apparent savings in learning across the modality transition. Figure S4 could easily be excluded and described in the methods. Figure S5 is potentially interesting, but the authors should be clear on what data is being modeled here. Does this combine easy/hard and pro/anti-saccade data? Why combine here?

Thanks for these comments. We think that Figures S1 and S2 (now correctly labelled as 5-1 and 5-2) are useful and should stay in the manuscript, as they show that our observation that statistical knowledge is largely not transferred across the modality switch also holds when an additional measure of participants’ decisions, namely their confidence, is tested. We agree that the results depicted in Figure S2 could be only reported, not shown, but we think that the Figure is useful as one can directly compare it to what is shown in Figure 5C-E (the same comparison, but for absolute angular error, not confidence). One can for example observe that the confidence rating seemed to be much more variable in trial 11 (Fig 5-2 A), compared to the absolute angular error (Fig 5C). We included however the information on the summary statistics, shown in Figure 5-2, which was previously missing in the result section of the main text (line 555-557).

We agree that Figure S3 (now correctly labelled as 5-3) shows similar results to what has been shown in the main manuscript, but that is exactly the point of this supplementary figure, as we were testing whether the results hold in another experiment (experiment 2), where more explicit and reinforced instructions were provided to the participants that trial 1-10 and 11-20 belong the same hidden target location.

Figure S4 is now excluded and we instead report which trials were included in the methods section (line 257-262).

In Figure 4-1 (previously Figure S5), we tested whether the results obtained in Figure 4E-F are confounded by the fact that previous guesses and observed hints could covary but were included in the SAME model, thus potentially leading to ambiguous results (as now explained in lines 501-502). For this reason, we repeated the modelling while fitting two separate models, either only including the previous guesses (Fig 4-1 A and D), or the previous hints (Fig 4-1 B and E). This showed that the model including previous guesses outperformed the model including previous hints in all subjects (Fig 4-1 C). For the analysis presented in Figure 4-1 data was pooled from all experimental conditions (easy/hard and pro/anti), as Fig 4E-F already showed that there is little difference between pro- and anti-saccades, and an additional extended figure (Fig 4-5A) concluded the same for the hard/easy task condition. This information is now added to the figure legend
