# Supplementary figures and images for: Previous Motor Actions Outweigh Sensory Information in Sensorimotor Statistical Learning
Source: eNeuro. 2021 Sep 28;8(5):ENEURO.0032-21.2021. doi: 10.1523/ENEURO.0032-21.2021 (PMC8482855; doi:10.1523/ENEURO.0032-21.2021)

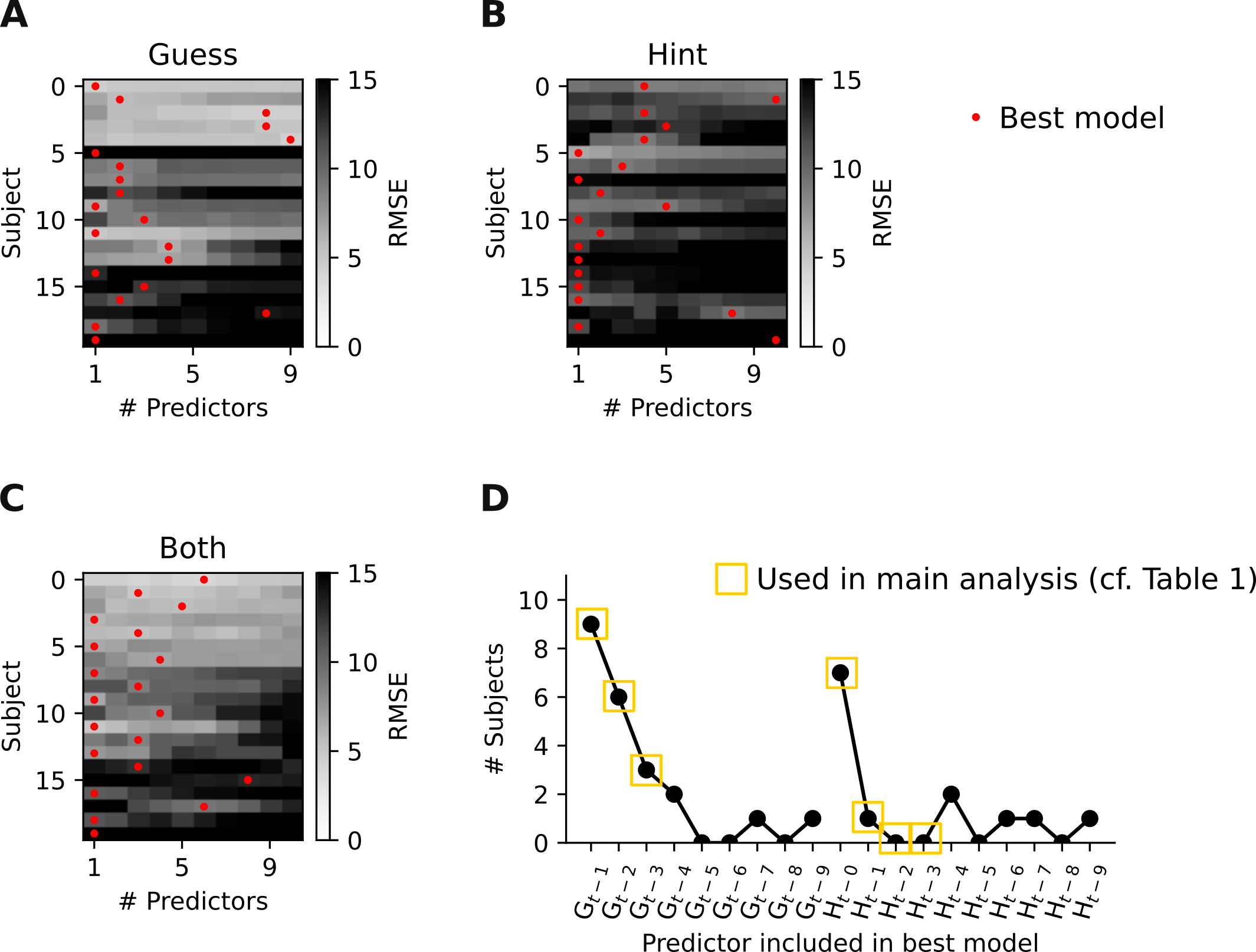

Supplement: Extended Data Figure 4-2 — Stepwise regression to select relevant predictors for model comparison. This figure is supplementary to Figure 4 in the main text. Here, we used stepwise linear regression (train function with method “leapForward” from the Caret package in R) to estimate the relevant number of past trials that should be included in our main model comparison analyses (Fig. 4; Table 1). We tried to predict the angular error in trial 10 from either all previous guesses (A), all observed visual hints (B), or a combination of both (C, D). Shown is the cross-validated fit accuracy (10-fold), measured as the root-mean-squared error (RMSE). Thereby, we found for each individual subject the number of trials (=predictors) that were included in the best fitting model (red dot), indicated by the lowest RMSE. To identify not only the number, but also the type of information that best predicted performance in trial 10, we analyzed which timesteps of previous guesses and current and previous hints were included in the best combined model (C) and created a histogram indicating for how many subjects the specific predictor (either guess: G or hint: H) was included in the best model (D). Using this approach, we found that for most subjects a model with six or less predictors, including a combination of previous guesses and visual hints, is best in predicting the angular error of the current trial. Thereby, we focused our main analysis (Fig. 4; Table 1) on three timesteps in the past (up to t-3). This allowed us to predict not only the behavior in trial 10, as done here, but also the behavior from trial 4 to trial 10. A general trend in the main analysis, which is also apparent here, is that previous guesses are better in predicting current behavior, compared to current or previous visual hints [lower errors for models including guesses (A) compared to hints (B)]. Download Figure 4-2, TIF file. [file enu-eN-NWR-0032-21-s03.tif]

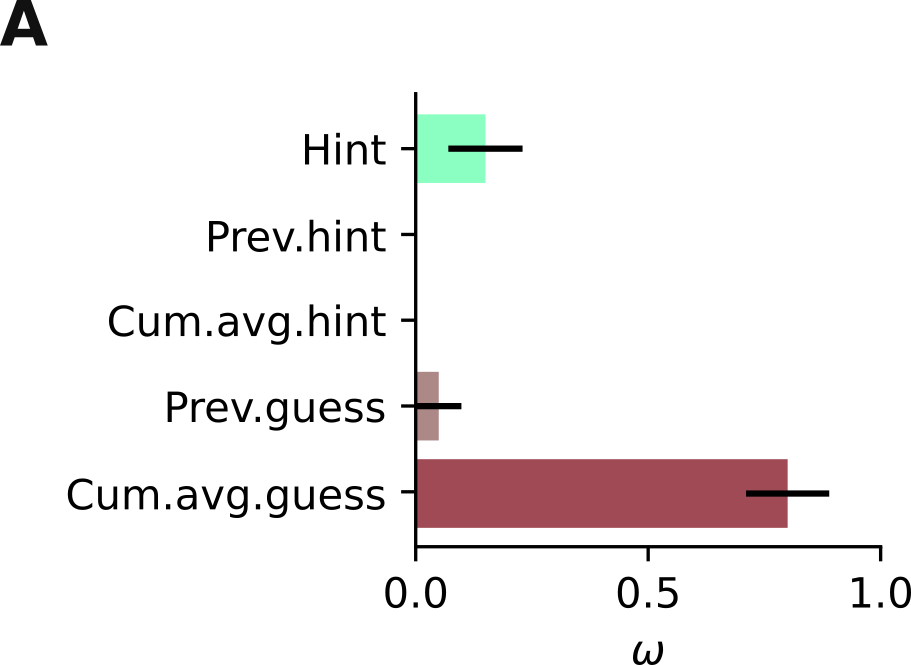

Supplement: Extended Data Figure 4-3 — Model comparison results of single predictor models tested on trials 2–10. This figure is supplementary to Figure 4B in the main text. Our main modeling results were based on models that included up to three timesteps in the past (n = 3), where n was determined based on a model search approach (Extended Data Fig. 4-2). To this end, we included trials 4–10, allowing us to test the models on a consistent dataset. To test whether our results hold when we include earlier trials in a block, we repeated our analysis focusing only on single predictor models (Table 1, models 1–5), which allowed us to use the data from trial 2 to trial 10. Similar to our main results, the best single predictor model was the one based on the cumulative average of past guesses. Download Figure 4-3, TIF file. [file enu-eN-NWR-0032-21-s04.tif]

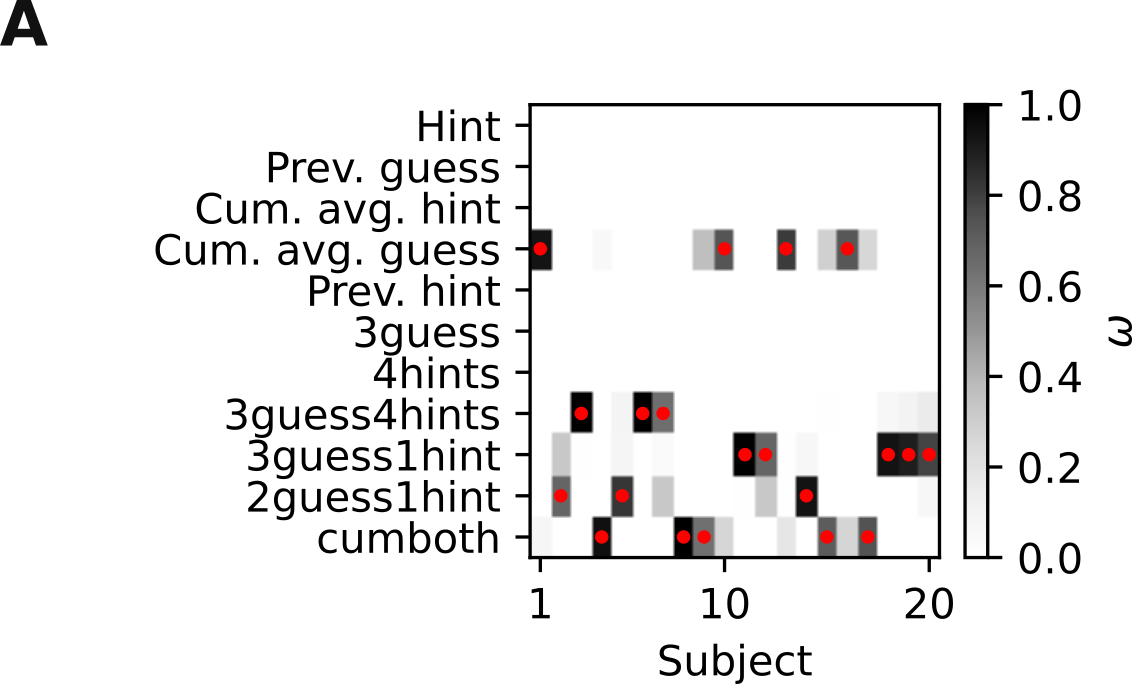

Supplement: Extended Data Figure 4-4 — Model comparison results for single subjects. This figure is supplementary to Figure 4B in the main text. Here, we show the model comparison results for each single subject individually. Download Figure 4-4, TIF file. [file enu-eN-NWR-0032-21-s05.tif]

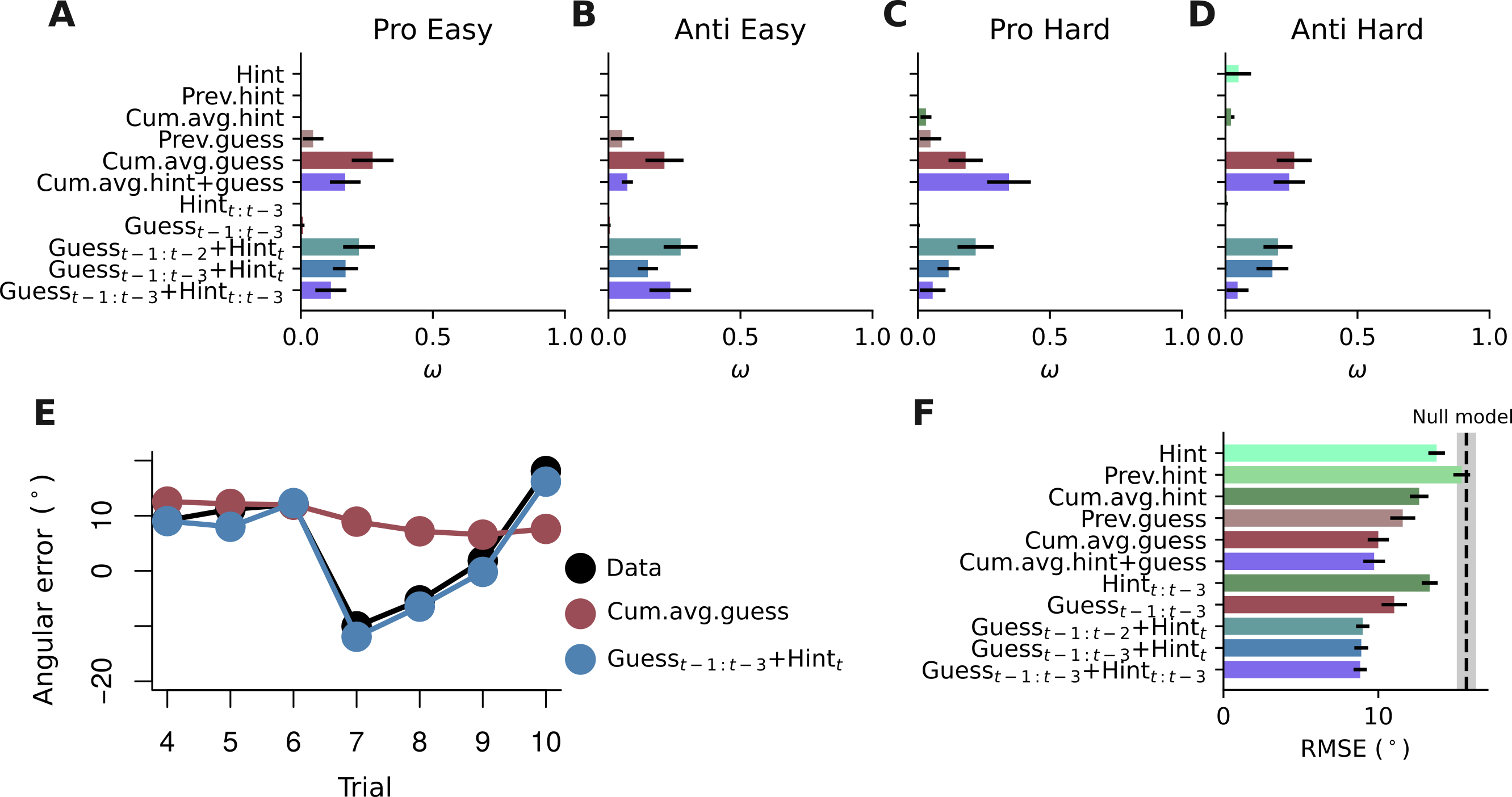

Supplement: Extended Data Figure 4-5 — Dependency of model comparison results on experimental condition. This figure is supplementary to Figure 4 in the main text. A–D, Model comparison results for data split according to pro-/anti-saccades and easy/hard task difficulty. Overall model comparison results did not depend on the response type or task difficulty. E, Example model prediction for a held-out data block. Data were pooled across experimental conditions, identical to the procedure used in Figure 4. F, Summary of model performance on held-out data, calculated using 10-fold cross-validation and the Caret package in R. Data were pooled across experimental conditions, as in Figure 4. Model performance was measured as the root-mean-squared-error (RMSE) for the difference between the true and the predicted angular error in the held-out data. The null model (also in the Caret package) corresponds to not using any predictor, but only fitting the intercept. Download Figure 4-5, TIF file. [file enu-eN-NWR-0032-21-s06.tif]

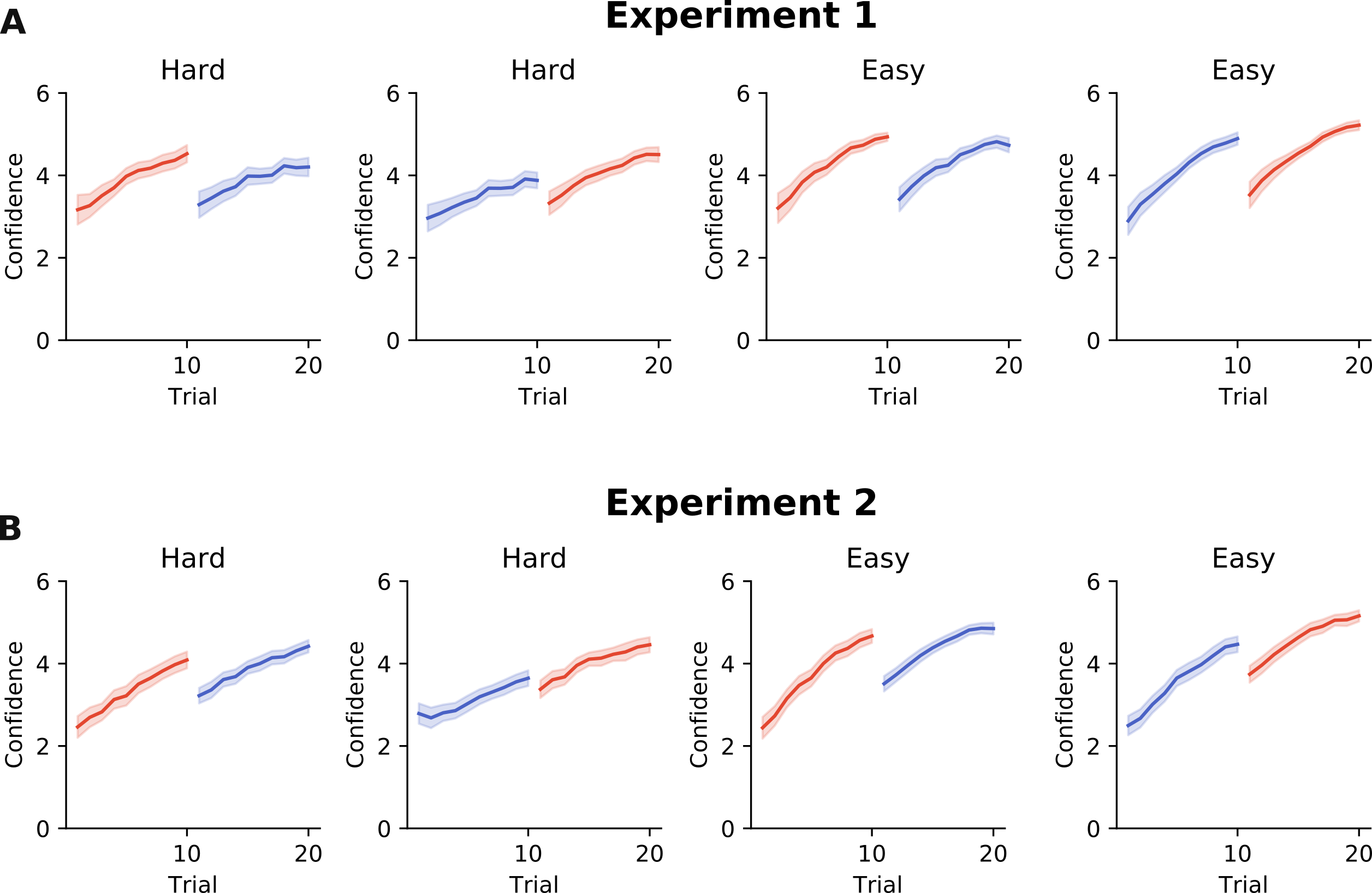

Supplement: Extended Data Figure 5-1 — Time course of confidence rating. Download Figure 5-1, TIF file. [file enu-eN-NWR-0032-21-s07.tif]

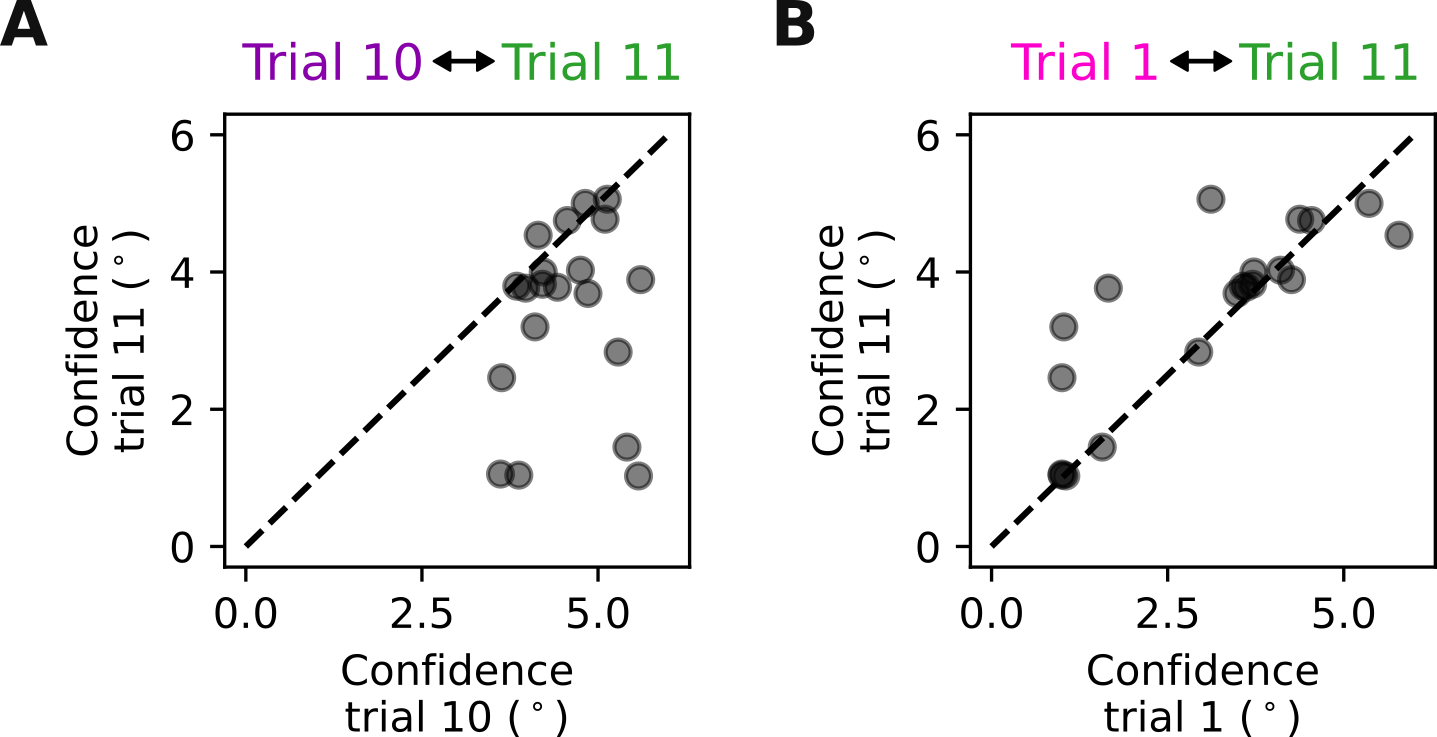

Supplement: Extended Data Figure 5-2 — Confidence drops after response switch. Figures 5-1 and Figures 5-2 are supplementary to Figure 5 in the main text. The confidence ratings demonstrated the same results as observed by analyzing the absolute angular errors of the eye movements, as there was a decrement in confidence from trial 10 to trial 11 (as shown in Extended Data Fig. 5-1A for experiment 1 as well as in Extended Data Fig. 5-1B for experiment 2), in all experimental conditions. The drop in confidence between trial 10 and trial 11 was significant (Extended Data Fig. 5-2A; paired t test; t = 3.67, p = 0.0016, N = 20). The confidence of trial 11 was not different from trial 1 (Extended Data Fig. 5-2B; paired t test; t = –1.78, p = 0.0906, N = 20). These results support the observation that after a switch in response modality, learning starts from an almost naive level. Download Figure 5-2, TIF file. [file enu-eN-NWR-0032-21-s08.tif]

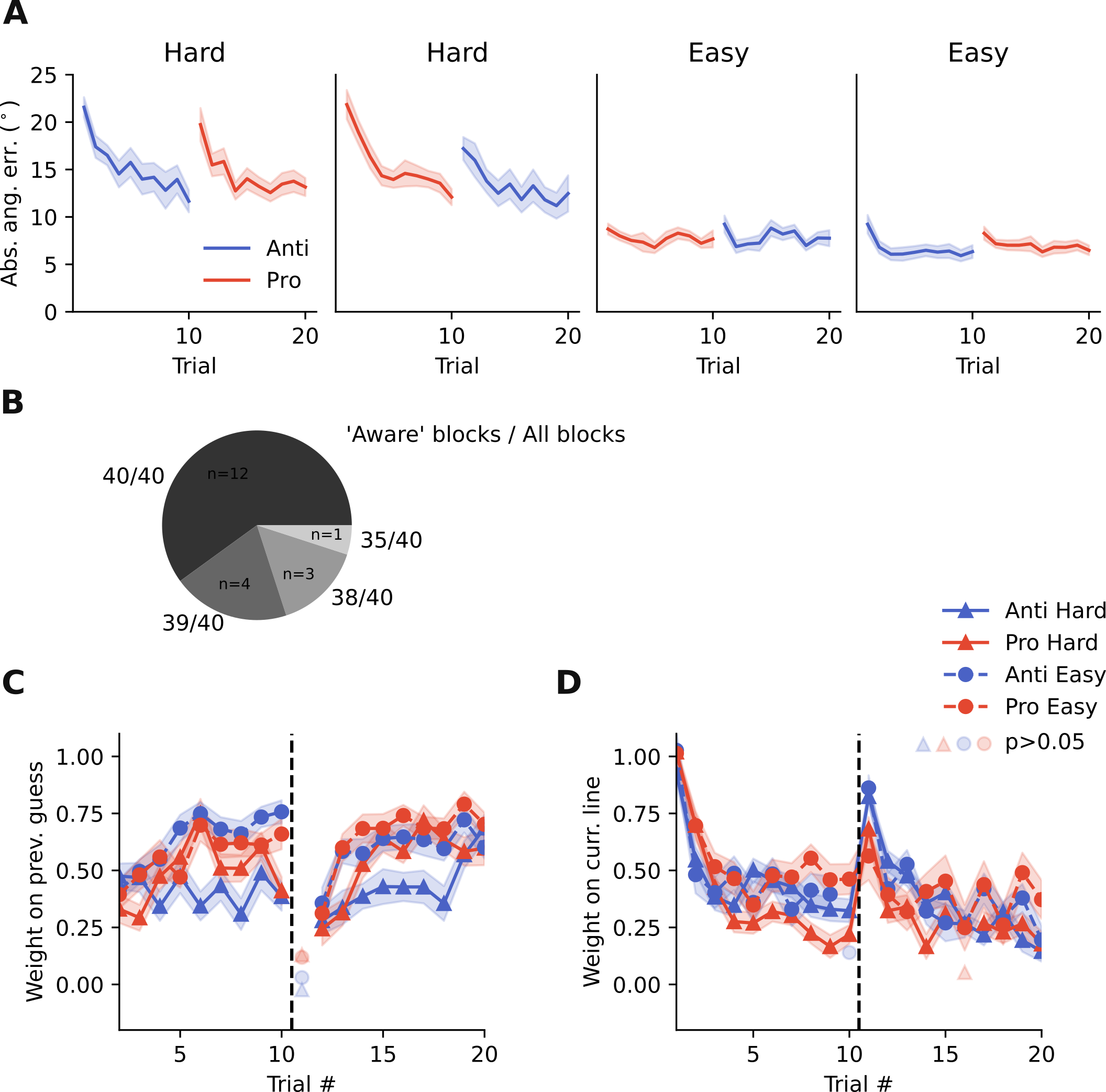

Supplement: Extended Data Figure 5-3 — Second experiment with reinforced instructions shows similar results. This figure is supplementary to Figures 5, 6 in the main text. These results demonstrate that we obtained similar results when participants were explicitly instructed that the location of the hidden target remained the same after a switch. Additionally, participants had to report whether they were aware of this rule, thus reinforcing the instructions. A, Also, in this experiment, performance dropped to almost naive levels after a switch in response type. B, The majority of participants reported to be aware of the rule. C, The weighting of previous guesses dropped between trial 10 and trial 11 (when the switch occurred), and instead (D), more weight was put on visual hints. Download Figure 5-3, TIF file. [file enu-eN-NWR-0032-21-s09.tif]

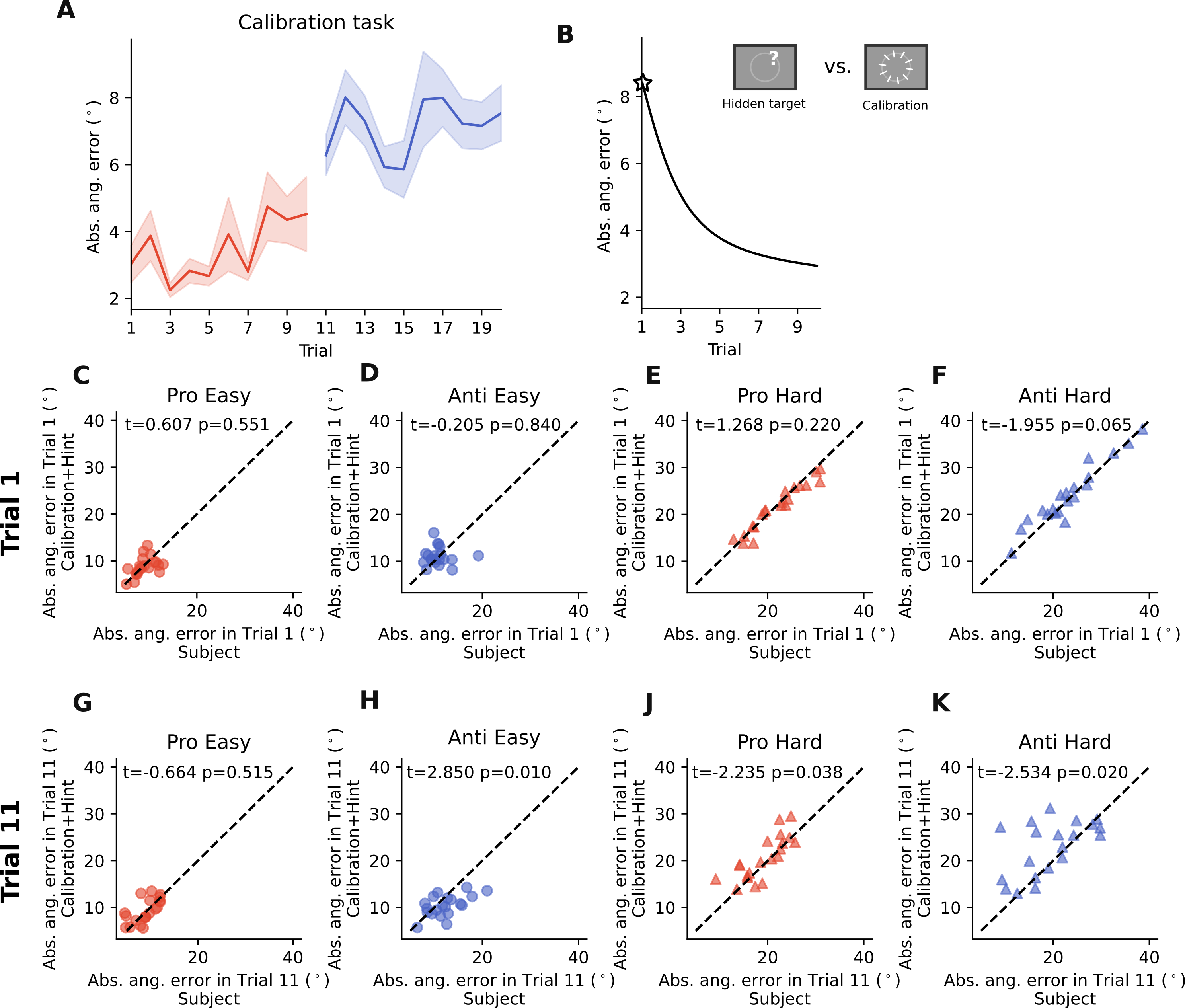

Supplement: Extended Data Figure 5-4 — Motor error estimation. This figure is supplementary to Figures 3, 5 in the main text. Here, we tested whether our assumption that participants’ estimation error in the hidden target task calculated as a combination of two independent sources of uncertainty, i.e., the motor noise (estimated from the calibration task) and statistical uncertainty (estimated from the distribution of visual hints in the hidden target task), was plausible. A, Time course of motor error in the calibration task. B, In the following, we examined trial 1 of the hidden target task and compared subjects’ actual performance to the theoretical prediction of adding motor noise and statistical uncertainty. If our assumption that both noise sources are independent and thus can be added is plausible, we would expect that the actual (x-axis) and predicted (y-axis) data would match. C–F, Results for all four experimental conditions. Each dot represents one subject (N = 20). Paired t tests were performed to test whether there is a significant difference between the theoretical prediction and the actual data and results are shown in the respective panels. Download Figure 5-4, TIF file. [file enu-eN-NWR-0032-21-s10.tif]
